# Moral Injury and Recovery in Uniformed Professionals: Lessons From Conversations Among International Students and Experts

**DOI:** 10.3389/fpsyt.2022.880442

**Published:** 2022-06-14

**Authors:** Jonathan Jin, Kyle Weiman, Suzette Bremault-Phillips, Eric Vermetten

**Affiliations:** ^1^Department of Psychiatry, University of Alberta, Edmonton, AB, Canada; ^2^Heroes in Mind, Advocacy and Research Consortium, Faculty of Rehabilitation Medicine, University of Alberta, Edmonton, AB, Canada; ^3^Department of Occupational Therapy, Faculty of Rehabilitation Medicine, University of Alberta, Edmonton, AB, Canada; ^4^Department of Psychiatry, Leiden University Medical Center, Leiden, Netherlands

**Keywords:** moral injury, PTSD, recovery, military, uniformed personnel, moral dilemmas

## Abstract

**Introduction:**

In the course of service, military members, leaders, and uniformed professionals are at risk of exposure to potentially morally injurious events (PMIEs). Serious mental health consequences including Moral Injury (MI) and Post-traumatic stress disorder (PTSD) can result. Guilt, shame, spiritual/existential conflict, and loss of trust are described as core symptoms of MI. These can overlap with anxiety, anger, re-experiencing, self-harm, and social problems commonly seen in PTSD. The experiences of General (retired) Romeo Dallaire and other international experts who have led in times of crisis can help us better understand MI and recovery.

**Objectives:**

In honor of Dallaire, online opportunities were created for international students and leaders/experts to discuss topics of MI, stigma, and moral codes in times of adversity as well as the moral impact of war. We aimed to (1) better understand MI and moral dilemmas, and (2) identify key insights that could inform prevention of and recovery from MI.

**Materials and Methods:**

Webinars and conversations of 75–90 min duration on MI and recovery were facilitated by Leiden University, the University of Alberta and the Dallaire Institute for Children, Peace and Security between General Dallaire, world experts, and graduate students. Sessions were recorded, transcribed and thematically analyzed with NVivo using standard qualitative methodology.

**Results:**

Ninety four participants engaged in conversations. Student engagements were attended by participants [*N* = 51; female (29), male (22)] from the Netherlands and Canada. Conversations were held with international experts [*N* = 43; female (19) and male (24)] from North America, Europe, Australia and the global south. Themes included: (1) recognizing the impact of exposure to PMIEs, (2) reducing stigma around MI, and (3) embracing the spiritual depth of humanity.

**Conclusion:**

Exposure to PMIEs can have devastating impacts on military members, leaders and other uniformed professionals. This may lead to development of MI and PTSD. Recognizing MI as honorable may reduce stigma and psychological harm, and facilitate help-seeking among uniformed personnel and other trauma-affected populations. Salient efforts to address MI must include use of accurate measurements of MI and integrated holistic therapeutic approaches, inclusive of spiritual and social components. Urgency remains regarding the prediction, identification and treatment of MI.

## Introduction

Military leaders and uniformed professionals require moral courage to handle the various types of dilemmas they often face (1–4). Dealing with moral dilemmas and showing moral competence (i.e., appropriately recognizing situations that require a moral response) is not an easy task within the culture of a military organization (5, 6). Moral dilemmas and associated moral decisions (i.e., a choice that is linked with an ethical value system) require weighing right and wrong against some type of personal or societal value system (7), as described by a Senior Canadian Forces Commander: “[A] lose-lose situation, a wrong-wrong. It’s where no matter what you decide to do someone is going to die. And you’re basically confronted with choosing the lesser of two evils. And that puts you into an enormous ethical dilemma and enormous stress” (8). Exposure to potentially morally injurious experiences (PMIEs) (experiences that violate one’s moral code) can result in significant harm and distress, much of which is purported to come from decisions that need to be rapidly made in the context of PMIE exposure. Leaders can deal with such catastrophes (e.g., Srebrenica and Rwanda) in a variety of ways which, in turn, can lead to differential later life health outcomes (9).

The toll of moral injury (MI) associated with making difficult moral decisions (10, 11) is significant. Notably, facing and making difficult decisions can play a role in the development of post-traumatic stress disorder (PTSD) and MI - the persistent distress that individuals may develop when exposed to events that involve perpetrating or witnessing actions that violate one’s core beliefs (12), or experiencing betrayal (13). MI is characterized by guilt, shame, intrusive thoughts, anger, and self-condemnation, a violation of a person’s core values/beliefs, and a shattering of the person’s core self (14). In PTSD this may be anxiety, anger, re-experiencing, depression, self-harm, and social problems. While not currently a recognized mental health disorder in the DSM-5, the phenomenon of MI is garnering much international attention (15).

The importance of awareness of the current situation is illustrated by a 19.1% prevalence of reporting a mental health problem in service members after returning from deployment to Iraq (16), and other significant prevalences have been reported by returning forces (17–19). Service members meeting criteria for probable mental health disorder and post-traumatic stress disorder (PTSD) were more likely to report problems with perceived or internalized stigma and barriers to care compared to those without a likely mental health disorder (20). There is clear evidence of the high cost of making difficult moral decisions and signals an urgent need for exploration of types of events that veterans perceive as morally injurious (21). It also underscores the need for reparative measures for the lasting impact of ethical dilemmas as well as moral injuries in crisis situations for military officers as well as leaders (22). Moral competence can be described as the ability to recognize that a situation requires a moral response, the ability to decide which of a range of moral responses is appropriate, and the ability to make moral evaluations of one’s own and others’ characters and actions (23). Like a navigational compass, a moral compass can become uncalibrated, causing deviation from “True North” or, as it applies to leadership, can compromise moral behavior (24).

One example of how MI can affect the lived experience of a leader is General (retired) Romeo Dallaire, who was the Force Commander of the United Nations Assistance Mission for Rwanda during the 1994 genocide. Military leaders such as General Dallaire are increasingly more likely to be exposed to PMIEs during deployment, as compared to life-threatening situations (20).

We currently lack approaches to tackle these imprints of MI that can serve as chronic drivers for PTSD, depression and also other behavioral problems, including suicidality. Current evidence-based interventions for PTSD focus on fear and emotional dysregulation, but less emphasis is put on factors that comprise the core of the MI, e.g., shame and guilt.

In terms of approaches to treatments, North Atlantic Treaty Organization (NATO) partners have been investing resources to develop novel treatment options for service members. Several gaps exist in current care provision for morally injured veterans, including issues related to holistic approaches, spirituality, employment and family functioning, which could ultimately improve veteran well-being (25). Elucidating the neural correlates of MI could be invaluable in guiding development of successful therapies. Of particular interest may be spiritually-integrated psychotherapies, pastoral counseling, but also virtual-reality therapies as well as the newer psychedelic therapies. Research into community-based, socially-oriented and culturally-specific approaches to MI are also required. Direct participation from chaplains, clergy, and religious communities in the treatment of MI may help address the spiritual issues (26, 27). A recent review concluded that veterans’ spiritual well-being should be routinely assessed and targeted in treatment (28). Interdisciplinary approaches are considered for future interventions with chaplain integration post-combat, which may help to increase continuity of care. In clinical populations, the use of clinical care in conjunction with peer support yields better treatment outcomes than either alone (29).

The scope of the present paper is understanding advancements in MI research and recovery. To increase our understanding in this topic area, this effort used online conversations and thematic analyses of qualitative data collected from university students, military leaders and clinical and political experts. They gathered on a series of online webinars and engaged in exploring areas of MI and recovery in uniformed professionals. A total of 11 online conversations were held, centralized around the lived experience of General (retired) Romeo Dallaire who was a thought leader in these discussions. We feel that the lessons from General Dallaire’s lived experience convey a message that extends beyond borders and has relevance for not only military populations but civilians worldwide.

## Materials and Methods

Qualitative thematic analysis (inductive and deductive) was conducted of transcribed video and audio recordings of General Romeo Dallaire’s discussion with three 75-min online conversations held between April, May, September 14, 2021 with graduate students (*n* = 48) from the University of Alberta and Leiden University (see Supplementary Material for transcripts) as well as eight 90-min conversations between September 22-October 28, 2021 from an expert panel of 46 leaders (11 military leaders, 35 civilian leaders) on topics of embracing humanity, moral leadership, moral courage, injury and repair; ethical decisions and moral dilemmas, intergenerational trauma, destigmatizing the impact of trauma and PTSD, engaged leadership and transformative culture change, re-envisioning leadership, and a pathway forward.^1^ Participants who reflect various and diverse perspectives were intentionally invited to engage in the discussions with the aim of ensuring an equity, diversity, and inclusivity (EDI) lens.

The recordings were transcribed by research team members and then thematically analyzed using Braun and Clarke’s methodology (30). Qualitative data analysis software NVivo (version 12.5.0.815, QRS Int., Melbourne, VIC, Australia) was used to facilitate flexible identification, analysis and reporting of themes in detail. Initial coding of the transcripts was completed independently by team members (JJ, KW) using an inductive approach. This ensured conformability, inter-rater reliability and bracketing of researcher bias (31). Inductive open codes were then combined into preliminary themes. A larger team (EV, SBP) were engaged in the secondary round of analysis focused on deductive coding informed by this paper’s objectives. Regular meetings were scheduled to provide opportunities for discussion, code verification, resolution of discrepancies, and determination of final themes with supporting quotes. An audit trail inclusive of memos and discussion minutes was used to review and examine decisions regarding primary themes and maintain credibility and rigor regarding the thematic descriptive analysis (32). In accordance with the local legislation and institutional requirements, ethical review and approval was not required for the study on human participants. Instead, verbal consent was received from all key stakeholder participants.

## Results

### Quantitative Descriptive Analysis

A total of 94 participants contributed to the data represented in this thematic analysis. Student engagement sessions (*N* = 3) were conducted on April 21, May 5 and September 15, 2021 and Critical Conversations (*N* = 8) on September 22, 29, 30, October 13, 14, 27, 28 and November 10, 2021. Demographic characteristics of student engagement participants (*N* = 51) and critical conversation participants (*N* = 43) are presented in Table 1 and highlight the level of expertise from which the data reported on in this manuscript is drawn. Female (*n* = 29; 56.9%) and male (*n* = 22; 43.1%) participants from the Netherlands (*n* = 14; 27.5%) and Canada (*n* = 37; 72.5%), and affiliated with various health sciences, law, native studies, computational psychiatry and education post-secondary programs, took part in the student engagements. International experts [*N* = 43; female (*n* = 19; 44.2%) and male (*n* = 24; 55.8%)] engaged in the conversation series were from a wide geographic representation and range of sectors, and served in diverse present and past leadership roles at local, national and international levels.

**TABLE 1:** Participant demographics (*N* = 94) for students engagements and conversations.

Student engagements (*N* = 51)							

Sex *n* (%)		Geographic representation *n* (%)		Faculty/Department *n* (%)		Academic level *n* (%)	
Female	29 (56.9)	Netherlands	14 (27.5)	Animal science	2 (3.9)	Bachelors	3 (5.9)
Male	22 (43.1)	Canada	37 (72.5)	Computational psychiatry	1 (2.0)	Masters	21 (41.2)
				Education	1 (2.0)	PhD	17 (33.3)
				Global affairs	4 (7.8)	PDF	3 (6.5)
				Kinesiology	1 (2.0)	Honorary PhD	1 (2.0)
				Law	3 (5.9)	Medical Doctor	1 (2.0)
				Medicine	2 (3.9)	Academic/Clinician-	5 (9.8)
				Native Studies	1 (2.0)	Researcher	
				Neuroscience	1 (2.0)		
				Nursing	1 (2.0)		
		
		**Institutional affiliation *n* (%)**		Occupational Therapy (OT)	12 (23.3)		
		
		LeidenU	14 (27.5)	Political Science	4 (7.8)		
		UAlberta	33 (64.7)	Psychiatry	9 (17.6)		
		UCalgary	1 (2.0)	Psychology	7 (13.7)		
		ULethbridge	1 (2.0)	Rehab Medicine	1 (2.0)		
		WesternU	1 (2.0)				
		Dallaire Institute	1 (2.0)				

**Critical conversations (*N* = 43)**							

**Sex *n* (%)**		**Geographic representation *n* (%)**		**Past and present sector affiliations *n* (%)**		**Past and present roles *n* (%)**	

Female	19 (44.2)	Africa	1 (2.3)	Academic/Research	33	Advisor	18
Male	24 (55.8)	Argentina	1 (2.3)	Civil service	5	Advocate	17
		Australia	1 (2.3)	Defense	31	Attorney	6
		Canada	21 (48.8)	Indigenous-related	4	Civil Servant	5
		Ethiopia	1 (2.3)	NGO	1	Dignitary	4
		Ireland	1 (2.3)	Private sector	4	Director	7
		Morocco	1 (2.3)	Veterans affairs	4	Health care professionals	15
		Netherlands	10 (23.3)			OTs	2
		Sudan	1 (2.3)			Pediatrician	1
		United States	5 (11.6)			Physician	1
						Psychiatrists	8
						Psychologists	2
						Traumatologist	1
						Military	31
						UN	11
						NATO	6
						Generals	3
						Colonels	5
						LColonels	1
						Captains	4
						Security strategist	1
						Researchers	30
						Spiritual care professionals	4
						University administrators	6
						Veterans	13
						Veterans affairs	4

### Thematic Analysis

Engagements between Dallaire, guest speakers, and students across numerous discussions yielded several major themes. These included (1) recognizing the impact of exposure to PMIEs, (2) reducing stigma around MI, and (3) embracing the spiritual depth of humanity. These themes, associated sub-themes are summarized in Table 2, presented in greater detail with supporting quotes in the following paragraphs, Tables 3– 5, and Supplementary Material.

**TABLE 2:** Themes and subthemes of the thematic analysis.

Themes	Sub-themes
Recognizing the impacts of exposure to PMIEs	Facing PMIEs and ambiguity of moral dilemmas
	Feeling the impact of a disrupted moral compass
Reducing stigma around moral injury	Breaking out of “old think”
	Moving toward treating with urgency
	Framing the injury as honorable (overcoming stigma)
Embracing the spiritual depth of humanity	Fostering resilience and interconnectedness
	Bolstering holistic approaches
	Leaving no one behind

**TABLE 3:** Additional supporting quotes for the theme “Recognizing the impact of exposure to potentially morally injurious events (PMIEs).”

Theme 1: Recognizing the impact of exposure to PMIEs	
Facing PMIEs and ambiguity of moral dilemmas	“There are young people in Canada, who have been child soldiers, and then they have immigrated to our country. And I had been doing some research, a few years back now, and interviewing some of those young people about their experiences, and what it was like to now live in Canada. And they would often say things like, “We would love to share our stories, but we’re not sure Canadians want to hear it.”” (CC 4).
Feeling the impact of a disrupted moral compass	“It was sort of like a decision of who will live and who will die? (…) But then, when you could hear the door being broken down, you could hear the machetes hitting and killing, the screaming, and the phone just dangling there. And those sounds. That’s when the shock of having to take such decisions will hit you. And ultimately, it’s the culmination of so many of such scenarios that start to eat away at you and your ability to sustain it. And in the end, I became overcome by so much of this, and felt that the demands on what you can provide, do have, at times, a limit.” (CC 1).

**TABLE 4:** Additional supporting quotes for the theme “reducing stigma around moral injury.”

Theme 2: Reducing stigma around moral injury	
Breaking out of “old think”	“When in reality what you see is [that] corporations invest so much – and families as well, and religion as well, and universities as well – we invest so much in conformity. So looking for people that look like us, that talk like us, that dress like us. And as soon as somebody actually dares to deviate, dares to be the misfit, the crazy one – we punish them for it. So I think, just overall, looking at group dynamics, and healthy groups, functioning groups, it is always about providing that little bit of oxygen for people to deviate, and not being punished for it.” (CC 7).
Moving toward treating with urgency	“We never use the term Moral Injury when it comes to traumatic experiences in children. And I think we should, because the worst trauma that children suffer from is interpersonal and I think it is usually moral. Somebody takes advantage of them, somebody fails to protect them.” (CC 5).
Framing the injury as honorable (overcoming stigma)	“And so that’s why we coined in 1997 uh after I went public the term uh operational stress injury because when we called it uh uh mental problem, or mental health, uh the soldiers didn’t want anything to do with it. An so you’ve got to frame this problematic of all these walking wounded that you have around you in as much as treating them with the respect, (…) that that injury is honorable and that they deserve all the care and the same concern and sense of urgency that you give to somebody who is physically injured to some who is mentally injured.” (SE 1).

**TABLE 5:** Additional supporting quotes for the theme “embracing the spiritual depth of humanity.”

Theme 3: Embracing the spiritual depth of humanity	
Fostering resilience and interconnectedness	“And as I mentioned earlier, in my opening remarks, making sure that they’re not just physically well-trained, but they’re mentally well-trained. (…) It’s not an invisible shield, and if I put my Kevlar on, my armor, my body armor, that doesn’t make me impervious to wounds, as General Dallaire was mentioning, impervious to moral injury.” (CC 2).
Bolstering holistic approaches	“On the one hand, his identity wounds would not have been so painful if he had not internalized certain beliefs about God and morality. On the other hand, these very same convictions also restrained his desire to act on suicidal thoughts and impulses, fueled his courage to pursue forgiveness, and mapped out practices and pathways for transformation that transcended my psychological interventions. Without attending to layers of spirituality and religion, in many people’s cultural identity, we risk oversimplifying the nature of our patients’ suffering, and risk excluding sources of resilience and strength that could be needed for moral repair to truly occur.” (CC 5).
Leaving no one behind	“The only reason I can now sleep (…) is because my wife is with me. There is a deep deep deep link between human beings that can be enormous saviour, because without that I was not going that way, in fact I was still going toward self destruction and yes my doctors had given me at the time, less than two years to live because I was literally working myself to death because I couldn’t handle the night. I could not handle the silence. That depth of that problem was not being resolved by the therapy or the medication it just lingered on.” (SE 2).

#### Recognizing the Impact of Exposure to Potentially Morally Injurious Events

PMIEs can have disastrous effects on a personal, social, and spiritual level. Sub-themes that were recognized include: “facing PMIEs and ambiguity of moral dilemmas” and “feeling the impact of a disrupted moral compass.”

##### Facing Potentially Morally Injurious Events and Ambiguity of Moral Dilemmas

Leaders are often faced with various types of moral dilemmas. On the military side, leaders experience tension between carrying out a mission and priorities about “[T]he people you are trying to protect” (SE 2). Limited resources can further complicate situations where leaders have to make moral decisions: “That’s what ultimately happened to us, is that we got a mandate on the [inaudible] there was no money and nobody was really interested in coming.” (SE 2). In some cases, choosing not to engage may be the best option: “[T]his was the example of my colleague in the United Kingdom, who could simply not accept that her advice on the legality on the use of force in the case of Iraq in 2003, was not followed. She found this unacceptable, and she had such strong feelings about this that her inner voice told her, “I need to resign, I cannot accept this.”” (CC 3). At the end of the day, it comes down to balancing the potential cost with the potential benefit: “I’d like to finish with a quote from former Head of the Department of Peacekeeping Operations, Under-Secretary General Jean-Marie Guéhenno. And he said, “In peacekeeping, you have a dilemma: To look the other way and have to live the rest of your life with maybe the notion that if you had moved in, you may have made a difference. Or, to move in, maybe with the risk of failure.”” (CC 1).

##### Feeling the Impact of a Disrupted Moral Compass

The consequences of facing moral dilemmas can be “[A] terminal injury, suicides were happening, families were being destroyed, women were being beaten, men were drinking, they were losing careers, they were in the streets. It was- it was quite, quite catastrophic.” (SE 1). The effects of dilemmas can have lasting effects as well: “[Y]ou’ll never erase the blood off your hands. It’ll, it’ll be hidden in the pores of your hands for life.” (SE 3). Part of the complexity of this lasting effect may be attributed to a deeper and more spiritual element that is affected with moral dilemmas: “Someone else is in pain, and we caused that pain, or we were unable to do something about it. What follows are tremendous feelings of guilt and shame and anger. What we call Moral Injury.” (CC 5).

#### Reducing Stigma Around Moral Injury

A need is expressed for a culture change to more effectively recognize and address psychological injuries, such as PTSD and MI, to overcome stigma as well as to move toward recognizing honorable injuries. Sub-themes include: “breaking out of “old think”, “moving toward treating with urgency,” and “framing the injury as honorable (overcoming stigma).”

##### Breaking Out of “Old Think”

It is imperative that all serving members affected by PMIEs be able to recognize their inner struggles and identify self-stigma that may impede/paralyze them in their ability to seek help. At its core, “Stigma creates holes in our life narrative. It makes us not understand how we got from there to here, and we don’t know how to get back.” (CC 6). The tone that leaders set in a community can open the door to reconciliation and healing: “I just wanted to comment, to be sure that we captured the important role of leadership in this task. That leaders establish the tone of communities, they say what is acceptable, and they also provide forgiveness.” (CC 6). For this reason, it is important to create a structure, organization, and society that is able to question non-inclusive practices: “That should never be. It should never be that we wait until an example of a human being or a system has failed – like terribly, that has turned into a crisis – in order for all of us to give us the capacity to question.” (CC 7). Traditions in a structure, organization, society should be critically evaluated because “Traditions are a value when they continue to assist humanity. When they are a constraint to human beings, no matter what group they are, they are of no use for the society. And that takes guts to do that.” (SE 1).

##### Moving Toward Treating With Urgency

A culture shift in moving toward treating moral injuries with urgency requires leaders who say “[I]ndividual justice depends to a huge extent on cultural justice. And that’s where moral leadership, I think, is absolutely essential, because in order to move communities toward those kinds of healing stances and actions, we need moral leaders to stand up to the flow in the other direction and say, “No, no. That’s justice, in that direction.”” (CC 5). One of the precursors to having leaders be more attuned to cultural issues is understanding that “[I]f they’re human, then fundamentally there’s equality. There may be nuances to it, and so on, and frictions. But they’re all human.” (CC 1). Once it is understood that all humans are humans, it bolsters the efforts to “[B]ring operational stress injuries into an honorable injury to be treated honorably and with the same urgency with physical injuries” (SE 2).

##### Framing the Injury as Honorable (Overcoming Stigma)

Overcoming stigma has multiple components associated: “So in order to deal with stigma, which is so prominent, I think we’re going to have to attack it in each one of these boxes. We’re going to have to attack the individual level, the cultural level, the systems level. We’re going to have to deal with how we stigmatize.” (CC 6). In terms of current efforts made, the framing of injuries as “operational stress injury” positively changed the help-seeking behavior in veterans: “When we called it an operational stress injury, and it was honorable to be injured in that way, it changed totally their philosophy in regards to getting help and support and so on” (SE 1). As well, tangible things, such as awards, have been a positive step forward in recognizing honorable injuries: “Tangible things like our Sacrifice Medal, where, if somebody has a psychological injury, they’re entitled to the Canadian purple heart because of this injury. So any opportunities we had to tangibly recognize these as injuries, I think it’s gone a long way. We’ve put in systems where we think about the multimodal way of treating this (…)” (CC 6).

#### Embracing the Spiritual Depth of Humanity

Integration of social and spiritual components need to continue to be bolstered in therapeutic approaches. Sub-themes include: “fostering resilience and interconnectedness” and “bolstering holistic approaches” and “leaving no one behind.”

##### Fostering Resilience and Interconnectedness

Open dialogue with individuals and communities that have been psychologically injured, such as some refugees and, globally, Indigenous communities, is an important preliminary step to healing: “But what we really need to do is to be having these open conversations, and this dialogue, with all of our non-indigenous communities and allies. So that we can all start to heal from the intergenerational traumas that continue to haunt our communities. Because wahkôhtowin. If we’re all related, then maybe what’s happening to me, is also happening to you.” (CC 3). It is crucial to inspire people to care about issues such as residential schools and the effects of moral injury as well as trauma: “Imagine what it is like for the parents who lost their children to [North American] residential schools. Imagine what it was like for the children who went to those schools to have lost their childhood.” (CC 8). One of the ways to move toward understanding and ultimately building resilience is having a spiritual depth: “The first is that there’s a lack of spirituality in humanity now. I speak not necessarily being focused on the religion but on a sense of inner spirituality of what the human being is. Because its not touched upon, its not nurtured, its not even spoken of, when you face these extraordinary challenges to every fiber of your being you realize that there’s nothing there behind you to be able to refer to re-establish a balance if you don’t have some sort of spiritual ability to communicate.” (SE 2).

##### Bolstering Holistic Approaches

The role of the spiritual dimension needs to be further bolstered since “Most of our empirically-supported treatments offer guidance for transforming painful states of anger, shame and guilt into more adaptive ones. Yet, for many persons around the world, forgiveness is intricately tied with spiritual beliefs, practices, and relationships.” (CC 5) and “[I]t’s widening the set of Best Practices that we truly need, to address Moral Injury.” (CC 5). The rationale for including spiritual dimensions for a biopsychosocial-spiritual approach is because love creates a spiritual depth that can alleviate destructive tendencies: “Only when you find some other human who can receive that can you alleviate that incredible destructive force that eating away at you year after year after year and making you worse” (SE 2). From a systems perspective, systemic barriers to health care access need to be addressed: “Just as mental health professionals too often exclude spiritual components of culture and intersectionality, systemic barriers to this type of community engagement too often limit our ability to address social and communal dimensions of moral repair. (…) We are at a point now where we need to better understand, access, and possibly enhance the healing potential of our patients’ social environments, and communities of choice. Without doing so, we risk perpetuating Moral Injury and not being as helpful as we possibly could be, as clinicians and researchers.” (CC 3).

##### Leaving No One Behind

No one can do it alone. Recognizing the need for formal therapy can help in the process of moral injury and PTSD recovery: “So in order to pursue with that it, it takes help. You won’t be able to do it alone. You need formal therapy to set up some parameters to help you both psychiatrists and psychologists who don’t always get along together.” (SE 1). Relationships can also be the motivating factor in a pathway to recovery: “So ultimately you know what saved me? I discovered love. I’ve finally found a higher calling in life. An actual loving of another human being and that love between my wife and I have now and myself was an element of backdrop to which all this other stuff could be adjusted.” (SE 2). The keys to effective peer support involve someone such as “[A] uncle a friend a colleague that’s willing to just keep their mouth shut and listen and let you empty 4 h worth of pain, that is an extraordinary tool.” (SE 2) as well as “[T]o just keep showing up, and to check in regularly, to keep showing up. And don’t underestimate the power of a compassionate, non-reactive presence in people’s lives.” (CC 5).

## Discussion

This paper uniquely engaged a convenience sample of international scholars, subject matter experts, leaders, and graduate students to engage in conversations on the topics of moral injury and repair. Romeo Dallaire’s experiences as a thought leader laid the foundations for these discussions. The impact of PMIEs was considered from a variety of perspectives and the need for further scientific research into MI was identified (12, 33, 34). The importance of bolstering ongoing efforts in the repair of moral injuries using a holistic, biopsychosocial-spiritual approach and imperative of reducing stigma emerged as central themes in our thematic analysis.

Recognizing the impact of exposure to PMIEs, its relation to PTSD and the process by which MI occurs was one of the key focuses of the conversations. Current findings in literature and recent studies provide evidence that events can be simultaneously morally injurious, traumatic and life threatening (35–37). Moreover, there is a strong intersection of PTSD and MI, as well as the association of MI with mental health outcomes to PTSD, including depression, substance abuse, addiction (38–40) and risk of suicide (41–43). Veterans with suicidal ideation and behaviors have shown higher levels of exposure to PMIEs (44). Such exposures can also have potentially disastrous effects on a spiritual level, and disrupt a person’s perspectives and moral compass (45, 46). These aforementioned notions link closely with and emphasize the first theme of our analysis, “recognizing the impact of exposure to PMIEs.”

As shown in our analyses, students and clinical experts accentuated the importance of advancing our understanding and assessment of MI. Access to assessment measures of PMIEs and MI was found to be essential. Various MI assessment tools to date have been developed [e.g., the Moral Injury Questionnaire–Military version (MIQ-M) (47), Moral Injury Events Scale (MIES) (48), Moral Injury Symptom Scale–Military version (MISS-M) (49), Expression of Moral Injury Scale military (EMIS-M) (50), and Moral Injury Outcome Scale (MIOS) (51)]. These validated scales aim to differentiate between subtypes of MI distinct from PTSD; most of these tools assess MI from a holistic biopsychosocial-spiritual perspective. They also have been used outside of the military, for instance with healthcare professionals during the COVID-19 pandemic (52, 53) and other populations (e.g., trauma- and disaster-affected refugees, Indigenous, first responders, healthcare professionals), and been developed for use in real world contexts. Other novel screening tools such as the Brief Moral Injury Screen (BMIS) have performed similarly to the aforementioned scales (54). In Nieuwsma’s psychometric study, it was found that individuals who failed to prevent or did something morally wrong had the highest symptom scores on measures of PTSD, depression, suicidality, alcohol abuse, and drug abuse, followed by those who reported solely witnessing a MI event (54). An international research consortium aims to develop and validate measures of MI as multidimensional outcomes to operationalize sub domains of impact and generate content for new measures of MI (51). Further research and implementation in clinical practice of these instruments is needed and clearly an area of rapid growth.

Reducing stigma around moral injury was also a prominent theme. This further complements literature reporting negative health outcomes associated with self-stigma such as PTSD (55). Dallaire, who as a leader publicly acknowledged his personal struggle with PTSD and MI, played a significant role in destigmatizing PTSD and MI on an individual, cultural and systems level. His vulnerability enabled a re-framing of the injury as honorable, thereby overcoming stigma. Dallaire’s disclosure of his struggle with PTSD and MI and depiction of his experience of the Rwandan genocide in “Shaking Hands with the Devil” (56) catalyzed a conversation about post-combat psychological injuries. His accounts contributed to lowering barriers to mental health care for military members, veterans and civilian populations. While significant barriers to accessing mental health treatment still exist across systems, peers and leadership, and internalized self-stigma levels have decreased, there remains a ‘hegemonic masculinity tenant’ (i.e., referring to dominant social positions of males) in the military (57). This poses a strong call to action to reduce stigma. Destigmatizing mental health treatment is not an easy task. It is felt that working with military members to understand their perceptions and attitudes, however, can help to change the discourse and facilitate a move in a positive direction.

The conversations also emphasized the need for targeted, evidence-based MI interventions. Use of psychotherapeutic approaches to MI by mental health professionals hold promise for improving the lives of many past and present military members. Our theme, “embracing the spiritual depth of humanity,” highlights the need for interventions from a holistic perspective. We see that addressing trauma among veterans and uniformed personnel has traditionally included front-line Cognitive Behavioral Therapy (CBT), Cognitive Processing Therapy (CPT), and Prolonged Exposure (PE) treatment (58). However, high non-response rates and persisting symptoms among military members and veterans post-intervention indicate the need for alternative treatment approaches (58, 59). Moreover, treatments need to address both PTSD and MI. Several MI interventions are in various stages of development and evaluation. Currently being trialed include Adaptive Disclosure Therapy (ADT) (60), Acceptance and Commitment Therapy (ACT) (61–66), the Impact of Killing in War (IOK) (67, 68), Trauma-informed guilt reduction therapy (TrIGR) (69), Spiritually-Integrated Cognitive Processing Therapy (SICPT) (70), Spiritually-Oriented Treatments (SOT) (14, 70–73), as well as therapies that focus on meaning-making (74), and use divergent thinking processes (75). Exploring and integrating deeper spiritual dimensions, existential realities, and change mechanisms such as self-forgiveness and self-compassion into traditional applications of CBT, CPT, or PE may be warranted and become more mainstream as studies emerge (28, 76, 77). These may be critical for effective treatment of MI. One recent systematic review of spiritual/religious-based interventions in veterans with PTSD found seven studies that reported significant improvements on PTSD outcomes (76). This would be a positive, culturally informed step forward in reference to the subtheme, “leaving no one behind” (77). Our findings in the main theme, “embracing the spiritual depth of humanity” further underscores the need for therapies to integrate the spiritual domain, with clinicians frequently describing that patients suffer from existential and spiritual conflicts as well as changes in beliefs about morality and humanity. As can be seen, there is an almost infinite array of possible directions and obstacles in future work on MI (78).

We recognize opportunities for MI that may be applicable to other populations outside of the military (e.g., trauma- and disaster-affected refugees, Indigenous, first responders, healthcare professionals) (79–81). The global pandemic, international tensions and threat of natural disasters has shone a light on moral struggles that are faced across humanity. In our conversations across multiple partners, some experts articulated that marginalized communities can be especially vulnerable to MI. In order to scale up more holistic MI treatment approaches, the impact of key health determinants, such as adverse childhood experiences, need to be measured and accurately assessed. Those affected by PTSD and MI would benefit from access to culturally relevant and personalized treatment approaches that promote resilience, meaning, purpose, hope, and social connection (82–84).

### Strengths and Limitations

This paper has some limitations. We believe that the people interviewed were carefully selected as they represented broad groups ranging from the military, mental health professions, legal domains, educators and academics. It is important to understand, however, that our sample is not representative of all veterans’ lived experience, since this population is highly heterogeneous, and the findings must not be overgeneralized. The student population was also not reflective of students across all academic faculties and departments. Yet, we believe that the unique nature of the present sample, including across generations of students and scholars, is inclusive of a range of areas of expertise.

### Future Directions

We feel the MI literature is rich and evolving and encourages conversations in this area. There is more to learn from global leaders who have faced PMIEs. Their insights can help us better understand MI and PTSD, identify preventative and treatment approaches, and means by which to destigmatize psychological injuries. The unique findings presented in this paper identify a number of important considerations. Future research would benefit from investigation of assessment and treatment approaches from a holistic and cross-cultural perspective. Inclusion of third wave behavioral and spirituality-integrated therapies is warranted. Our findings will inform key areas for future policy and practice recommendations, as well as directions for future research.

## Conclusion

This paper presents a unique series of conversations between graduate students and world experts around topics of MI and recovery. The results of these conversations may serve to forward the discourse surrounding PTSD and MI. Based on findings from a qualitative thematic analysis, it is evident that leaders often experience psychological injuries as a result of being placed in situations with moral dilemmas. The stigma associated with psychological injuries contributes to challenges with help-seeking among military members and veterans, and positive treatment outcomes. Recent efforts to expand more holistic, personalized and culturally-sensitive therapies to strengthen efforts in MI recovery have been encouraging. Urgency remains regarding the prediction, identification and treatment of MI.

## Data Availability Statement

The datasets presented in this study can be found in online repositories. The names of the repository/repositories and accession number(s) can be found in the article/Supplementary Material.

## Ethics Statement

Ethical review and approval was not required for the study on human participants in accordance with the local legislation and institutional requirements. Written informed consent for participation was not required for this study in accordance with the national legislation and the institutional requirements. Written informed consent was obtained from the individual(s) for the publication of any potentially identifiable images or data included in this article.

## Author Contributions

SB-P and EV conceived, designed, and conducted the manuscript. JJ and KW conducted the analysis. All authors drafted, revised, and approved the final manuscript submitted for publication.

## Conflict of Interest

The authors declare that the research was conducted in the absence of any commercial or financial relationships that could be construed as a potential conflict of interest.

## Publisher’s Note

All claims expressed in this article are solely those of the authors and do not necessarily represent those of their affiliated organizations, or those of the publisher, the editors and the reviewers. Any product that may be evaluated in this article, or claim that may be made by its manufacturer, is not guaranteed or endorsed by the publisher.

## Footnotes

1
https://www.universiteitleiden.nl/en/dossiers/the-university-and-the-war/cleveringa-chair/moral-courage-leading-in-times-of-conflict-and-crisis
