# Supplementary material for: Moral Injury and Recovery in Uniformed Professionals: Lessons From Conversations Among International Students and Experts
Source: Front Psychiatry. 2022 Jun 14;13:880442. doi: 10.3389/fpsyt.2022.880442 (PMC9237246; doi:10.3389/fpsyt.2022.880442)
Supplement: Supplementary file 1 [file Table_1.DOCX]

Supplementary Material

# Supplementary Data

**April 21, 2021 Session 1:37 duration**

Moderator 1: Alright good morning to you all entering this special session of today April 21, 2021. Good, good to see you all back from the ones that were with us yesterday if you uh were not with me yesterday but if you were me yesterday I can tell you that I my name is XXXX. I am a psychiatrist, a military psychiatrist at the um, um, um, military mental health services in the Netherlands and I am a professor of psychiatry at Leiden University in the Netherlands. I am in the Netherlands at this moment and local time here is 5 o clock pm and this is a wonderful session that I have been looking forward-

Unintelligible: 0.58

Moderator 1: very much…please mute your microphone. I have been looking forward to this very much because we have with us uh, the general Romeo Dallaire uh, who has reserved time to engage with all of us to speak about um, any topic that you want to bring to the table to him that um, refers to his career as um, as a leader and um, what I’d like to do to open up this session briefly to bring it into our awareness uh this is the opportunity that has started this initiative and actually it is the Cleveringa chair that was awarded to the General Romeo Dallaire in the last of November of last year and he presented uh because we were in COVID a speech with the title Are All Humans human? Ethical Responsibility into a New World Order. The University of Leiden awards uh, uh, uh, uh, a special person every year because of the Cleveringa chair that we have um, um for 50 years installed at the Leiden University. Some of you have already known this and it is a professorship on…let me summarize it as moral courage. Since Cleveringa was a professor of of the University of Leiden who stood up when our country was invaded by the Germans and uh this professorship is really about moral courage and this was awarded uh last year to Romeo Dallaire and in the um in the um in the in the year after that we wanted to leverage and create opportunities for Romeo Dallaire for engaging actually with uh with uh people uh that could benefit from his wisdom and his uh his experiences by um by organizing sessions like this. This is the first one that we have and Ill read just the back of this and then Ill give the word with great pleasure and honour to to the General himself. On the back of the book it says Canadian General Romeo dallier 1947 was a UN commander in Rwanda at the time of the genocide there, subsequently becoming the champion for human rights. He has worked as a researcher at several different universities and was a senator for Canada for 9 years. Dallaire’s 1997 revelation that he suffered from post traumatic stress disorder as a direct result of his mission for Rwanda paved the way for destigmatizing this potentially lethal operational stress injury amongst military veterans as well as for first responders. Now with this brief introduction I’d like to give the word to the General Romeo Dallaire. Seeing that we are now a little bit after 5 and the general has reserved time for about an hour and we may go a little bit over an hour but at the moment of the hour we’ll say hey how are we doing and we’ll see if we can stretch it, if we need to stretch it. So without further ado um, General Romeo and I didn’t say that the General was born in the Netherlands I said that yesterday. So with that cliffhanger [laughs] Romeo Id like to give it to you.

Romeo: Well thank you very much uh with my 27 cousins and god knows how many children uh uh and nephews that I have in Holland I have… return often and in fact um with my wife we returned last year for the 75^th^ anniversary uh and so I am very uh very uh proud to be able to hold the chair for a year and have already offered that uh at no cost I am prepared to keep the chair for another year [laughs]. In doing that humbly is kind of difficult for a general…I uh…am very interested in uh the breakdown of you students who are attending uh and I also…on some of the questions you were raising yesterday and I thought I would uh, uh give you a sort of a an intro to permit you to germinate questions that are more specific to you arena and interest because that’s where you feel you are actually gaining something personally and collectively uh from the uh the event. I uh we can’t necessarily negate which we find ourselves today with the COVID and the international response to that and how uh it has an interesting curve toward globalization uh and um and um some um some see of resurgence of sovereignty and nations just taking care of their own and self-interest, while others see a bit of an opportunity…could have been an opportunity for globalization goal for humanity and not just for the economics uh or some of the politic uh, uh interfaces. It is uh because of that uh and because of the fact that we are still stumbling through an international community uh that is trying to grapple with exactly where we find ourselves with humanity at this time since the end of the Cold War. Remember that we stumbled into the 90’s without any really comprehension of what might specifically happen uh…the big powers, the Eurocentric scenario sort of uh…it came to a near end uh then we saw an explosion of failing states and uh masses abused of human right, even genocides in a number of other nations and nation states uh who were now no more under the uh the XXX the big the big two blocks and uh they have been uh destabilizing their regions not only, their countries, their regions and creating horrific scenarios of human rights abuses and conflicts uh that has even gone into scenarios of generational wars by the use…with the massive use of children as weapons of wars that sustain their operations as they recruit them to be simply cannon fodder in man of these conflicts. So we stumbled into…but not into…George Bush thought that we would be going into world order. We stumbled in to world disorder because we were on job training, ad-hocing, um crisis managing, we were sending all kinds of resources uh security resources in particular, humanitarian resource sin other dimension in trying to put a cap on many imploding nations only to find that we are often too late uh and the subsequent catastrophe they create were massive both in the destruction of human beings in the humongous number of refugees that were displaced which in themselves are a source of enormous threat to other nations because those sites uh are easily expanded into pandemics um the rage of those who live their easily has turned to extremism that goes into violence and even terrorism. Diasporas who are moving massive around the world are also locked in a bit to what’s going on at home so they tend to even uh slightly destabilize the countries in which they find themselves as you experience and we experience. And so this whole framework of humanity is still in flux and COVID only proved uh that the international community has not been able to grapple with this from a global sense, from a humanity sense, but is still operating from the Westphalian nation state, sovereignty, and nationalism framework and as such at a time when all of humanity well can nearly be skyped by anybody in real time um we are still operating with old tools. This uh in my opinion is negating an enormous force that is in existence and in potential that has not come to the floor yet. And that is the youth of the world. The era where the youth are uh sort of side show uh sort of nice to have that we will have a couple uh token in in involvement, that era is over. We are in a revolutionary time frame regarding youth where on one side they’ve got all the tools now uh to operate globally with the communications revolution and in fact a number of them are global uh global when they play these damn war games so they they generation of the under 30s who are mastering more and more and maturing uh the social communications for the whole of humanity are in my opinion uh a leadership body that has an activist role that is far beyond any of the uh um generations before it because you go the tools to influence in real time throughout the world and secondary not only that you are uh able to communicate and influence uh and uh become strong and vibrant activists uh but you can also ultimately look at wanting to be held accountable for bringing reform and changes and revolutions in the institutions of the world, in the construct of how world bodies are adjusting to this new era of global humanity. My opinion is that this generation that I speak of is the generation without borders. It’s Its not looking locally. It doesn’t want to look locally and because it’s a generation without borders it is global already and because of that it can far more take on such subject as the environment. They understand the illogic of yeah a bit of oil and water and uh dirty water abuses in certain areas but they see a much bigger picture on the impact on humanity and in fact are able to articulate that uh because they can see all of humanity. They can talk to all of humanity, that they need to formulate a communion between humanity and the environment. They can bring the debate at a higher plane than what we are seeing at the generation of the sort of leaders of our time which are still locked into old things and old tools. I am firmly in belief that this uh belief that this generation without borders is prepared not only to engage deliberately and influence massively through the demographics in so many developing countries that they can overwhelm the populations to change the nature of politics and priorities. And so in my opinion the youth of the nation are looking not at how will humanity survive the future, btu are actually looking at how humanity will thrive in the future and if you are of the school in humanity trying to survive you’re already in a pejorative situation in regards to trying to bring reform and actually moving humanity. One must build a concept on which humanity will thrive. Now we could talk about uh, uh other uh arenas uh at the grander scale but I think there is another dimension that I would like to bring to your attention uh as uh the environment, the engagement of youth and the accountability of youth of being engaged. Accountability of youth being engaged. Uh, uh, the absolute criticality of the [inaudible] the equal of women and men. We have all our institutions from catholic church to god knows what institution, our philosophy of leadership in the world  has been based on male dominated thinking and because of that it is simply not meeting what the younger generation who are far more attuned to the realization that the quality of women and men are uh generating and reinforcing each other. That there is a synergy between how the women are thinking about leadership, how men think of leadership and that there should be a marriage, a sort of integration of the two thin- thinking. And because we are not getting that women equality and that women engagement, we men are going to continue to stumble in the old think of how we believe our philosophy of leadership is in fact the philosophy of the world. It is no more. And women must engage and must move the yardsticks into a new philosophical framework of leadership which drives ethics, which drives moral values, which drives the equivalency of humanity in regards to all humans being human. Who can be closer to life than a woman who ultimately gives life? And so in my opinion this other dimension is in critical requirement of being changed and the younger generation who have got to ram that down the throats of the generations ahead of them because if you don’t do it we’re simply going to keep moving in a male dominated world which has reached its peak. It just doesn’t have the new [inaudible] of what humanity is and what youth of humanity are seeing into the future. Now I’ve been informed also that there are subjects of great interest to you in regards moral dilemmas, um, um, peacekeeping um, the law, armed conflict, um the use of children as weapons of war, uh what is the leadership of tomorrow and I got to tell you the leadership of tomorrow has got to be either equal uh at the minimum uh of women and men but its got to be also framed in a new philosophical framework that doesn’t exist today. How moral injury affects us and how we react to that, how we sustain that, how do you, how do you, stay uh when you are yourself injured uh I think that uh these subjects are pertinent as well as how we can prepare people for crises as we are going through now and many in the future.

Moderator 1: General if I may interrupt uhm would you like to because you gave a very thought provoking uh uh um uh speech uh on the these topics that you just outlined. Would it be okay for you to ask to to invite or allow students to comment on it if you wish or would you like to unfold and progress to other topics that have been forwarded to you?

Romeo: I, I, I, am I am not well known for brevity. A retired general and a retired politician trying to be brief is just doesn’t exist. I wanted to just give this opening statement and enumerate some of the points that were raised and now the floor is open-

Moderator 1: Wonderful, Wonderful

Romeo: I am expecting an onslaught

Moderator 1: [laughs] We, we didn’t give a sort of an order that we’re going to do that. I didn’t call you by name yet but, but, but we have um we have um we have now 19 participants in this in this sort of classroom um dear General. And any how should we go about this I have not often moderated this session btu I think we could go by if you see my hand you could sort of wave your hand and I could look at you and-

Romeo: Yes

Moderator 1: yes okay please raise your question to the General. And we could section this out first because General outlined maybe some I don’t know may I may I call this sort of maybe you don’t like this word this feminization of society is that now—

Romeo: I have absolutely no problem with that because uh the reverse is what we’ve been living under. It’s been masculinization of socie- civilization so I have absolutely no problem with introducing that as an argument. Yes.

Moderator 1: Maybe I should give the rod by because yesterday I counted the number of males we had in our classroom and there was one male and now I see one two three males and the rest no 4 males if I see them. Any of the 4 males feel that they have an urge to respond to what the General just said, about the position of the men uh men as a representation of the youth but also as s as a gender specific person maybe uh XXX uh XXXX or XXX. Please open the mic. If you feel like hey give me a moment to reflect.

Student 1: Sure I’ll, I’ll, uh, opt in

Moderator 1: XXX XXX go ahead

Student 1: unequivocally yes, yes, my goodness yes [laughs] I am I am happy to uh like how do I say I am happy to platform other ideas that are not just white males again. Being in this position, being invited to this conversation I felt like a deep sense of privilege. The ideas that I am having about futures of humanity I am I am finding are repeated to have more of a diverse perspective, on that is more emphatic makes uh not only sense to me but a needed driver of what is next so a less of a question but more of like an unequivocal acceptance of the fact.

Moderator 1: thank you for sharing. XXX is a um, PhD student counselling psychology.

Student 1: mhm

Moderator 1Who wants to, any of the females, any of the other genders would like to respond back to what you just heard. Anything that you’d like to bring back to the general?

22:13

Student 2: I think my question might be more about how?

Moderator 1: XXX

Student 2: Yes [laughs] um so being a female and it being much harder so doing my Phd and it being much harder being in a male dominated society where the uh social standard and many times where you feel like the system has failed you? How do you keep fighting in the system when you feel betrayal by the system? If that question makes sense?

Romeo: I, I think that uh there has got to be a realization by the women and the young women in particular the, the, the, under 30 women or 30ish um right down into the schools systems. Uh that they are now at the cops of a revolution. You had the feminist movement and the feminist presence, you’ve had so much effort done by women trying to move into the male world and, and some has been very quite positive, however as my wife has just finished writing a book on um leadership without the armor which is which is leadership which has far more a feminine perspective into it. What is interesting is the fact that you are now at a point of imposing a new think into leadership. You’ve been you’ve’ been absorbing and reading and living what was going on. The women who have precede you so many have in fact turned into a bit like men. You know the leadership many of them are using are examples of male leadership that they have subtly made a few changes but ultimately they are they are very much sort of acting under that sort of discipline, that sort of thinking, that sort of process. That’s not at all helpful to the advancements of the two genders. So how do you do that is that you must take up a revolutionary guard type of process. Find yourself a red book like the Chinese did in that I suppose in one way or another. Become activists with the ability of the social communications to build momentum real time. Do not underestimate the power that you have in societies by mastering the social communications tool. Its still an immature system. Ill give you my example. I’ve been very much involved with child soldiers and I was involved in Uganda with Joseph XXX. Trying to go after get after that terrible man and with the Lord’s resistance army who was recruiting um uh uh illegally of course and abducting children. And so at one point uh the organizations states got a bunch of universities involved to raise funds to say we’ve got to stop this. They are abusing these children and lets make a big fundraiser. We are going to get the money and we’re going to go after him and we’re going to stop him. And they, they, went on social media and they collected 70 million bucks and they did all kinds of stuff to do that uh but ultimately they did not succeed because they, they, they, were not able to implement what they were talking about it. They were simply able to move people. What happened with tha is Coney became so known on social media that it enhanced his prestige in the world. He became to him he became nearly a god in regards to non XXX actors trying to uh take down other governments. So there is the way now. Now this era of this social communications and revolution that you can coalesce into your thing and you can bring power to your arguments. And you must absolutely fight the idea that you’ve got to adopt the male philosophy of leadership as the instrument for the future. It’s not. It is not. It is old think. It has got to be a far more integrated male-female philosophy in which you are bringing the new things to it and as my wife was uh explained in her programs, it is very interesting to see how the human being has returned into the leadership construct sort of like a reinforcing leadership from the people to the leader versus the leader down to the people which women are far more capable of handling. That’s my short version.

Moderator 1: so, so I saw XXX nod you were saying yes what the what the first section of the general’s comments was. Do you want to add why you nodded to what he was saying? He was saying like we need to become activists. Take the momentum right and then you, you, you were nodding very- So anything you wanted to add or ask XXX to that?

Student 3:  um I think what I was nodding so heavily at was the idea that women as leaders to day have become masculinized and um I do a lot of work on gender and I actually work in the area of child soldiers in Uganda actually and I’ve done a lot of work on gender in that area. Um and what I find so interesting is the idea divorcing masculinity and femininity from biological gender or biological sex and making it more a gendered thing and I don’t know that its necessarily so much women leaders that we need which of course obviously having women in leadership positions is wonderful, but having feminine knowledges in these leadership positions. I don’t think there is problem with having males and men in leadership positions provided that they are still running on this same XX ideas of power that have infiltrated IR and military for since Christ was a boy, but moving into these more feminine ways of seeing the world that is divorced from biological sex. So that’s what I was thinking about when I nodded so heavily. I do have questions for the general. Millions of them but I will save them later when we are in a more appropriate topic.

Romeo: Well I think uh just to follow you through. Uh and I hope you uh uh you know a bit of the work we are doing at my institute and out of XXX center for excellence. But what you’ve stated is quite correct uh recently we’ve have a raf- harassment and problems with very senior officers that we’ve moved aside and one of the solutions the government was to promote the women to a very senior position. This women comes from a system that is dominated by males and made her way up there because she was able to adapt to that. So she’s not bringing the real essence of women leadership. She’s brining a XXX of it. She’s bringing a element of it there’s no doubt. And so this tokenism of presentation of women is totally forced. That’s why there is no room anymore for some uh, uh lets see what we can do uh with women in different leadership roles, it is what is the new philosophy of leadership that is now an existence in the world that incorporates women and men’s skills, knowledge, experience, values, way of thinking, uh perspectives of life uh. We have had and I got…we’ve had women killed in operational theatres in combat arms in the combat forces. And uh we still have that adapted philosophy that incorporates women commanders with male commanders in order to bring a new philosophy of leadership. It’s still a male dominated one and that’s not going to survive. The young people will not survive under that. Young men will not join that and young men feel that its not what they’re learning in school and society. Where society is bringing them closer together. So what other big question did you have?

Moderator 1: There’s one male XXX. Please go ahead.

Student 4: Hello. Hi yeah. First of all I’d like to say how huge of an honor it is to be able to attend this meeting with you General Dallaire and thank you for such a lovely opening statement. I am in total agreement with your assertation that we as, as society need to make a measured effort to shift toward a more female dominated global structure. However I was wondering what you think what the best method would be from an organizational standpoint. I understand that certain fields an sectors are able to do it better than others but I see some fields which are still male dominated like tech, engineering and other STEM related fields, which seem desperate to entice more women to join but yet it seems to be an uphill battle. Do you have any suggestions for how these laggard fields and sectors can progress accordingly?

Romeo: And thank you for the question because I come from one that is uh military and any of the uniform structure be they policemen, be they fireman, be they uh, uh you know prison guards, be they bo- border guards, any of those uniform organizations are male dominated and women have been trying to break into them and as they do they get absorbed into that sort of male construct. And so there is nothing less than a than a deliberate action that the effort by women be presented from a different perspective from what society is expecting of the engineering profession , of the military profession, of that. And so those professions need to be reformed and uh those reforms have got to come from enlightened women and of course hopefully more enlightened men in coming to grips with finding what the ethos of their organizations. How is it make up? What are the parameters of their ethos? What are the parameters of who they really reflect? How do they really want to communicate their leadership competencies to a much more diverse, let alone gender uh mixed uh, uh following or, or troops, and so it is critical that institution be pushed by social uh, uh, uh, structures to demand that they conduct these massive reforms. I’ll give you…we were just talking to one of the persons whose right now involved with the current crisis in the Canadian forces. As I indicated to her I said there is no way around getting the leadership, the officer core, the ethos of the officer core and the military to change its perspective and attitudes towards women and abuse of women and and maltreatment of women, let alone the opportunity of women to fully prove their potential as women and not just as you know so… women working under male conditions. It, it cannot happen unless there is a deliberate action to reform that which means conduct a purge, conduct a, a philosophical realignment, now get industry to do that, get the professional orders to do that. That is what we’re going to try to do in the military and I think that’s what’s got to be done with other profession. Without that its it’s a sort of like attrition warfare. Women will continue to chip away at it while the guys continue to build a barrier. Change the nature of the beast. It is now the time and I I’ll emphasize it with the following: when the cold war ended we ended 300 years of classic warfare. It culminate in the most extensive classic war which was the cold war in which yeah we didn’t fire many shots but we certainly had nuclear weapons and massive use of millions of troops and equipment and all that kind of stuff and one side finally went to the other. But when we dropped that classic war framework and we looked at imploding nations and failing states, we discovered that there was nothing there for the security side to be able to be a value added to bringing not only peace, but preventing conflicts from happening. And that’s why you have a continuum still of this uh inability to get a handle of these conflicts because we’ve just not figured what are all of our parameters about it. I am a commissioner on an international conference on the principles of peace, lasting peace. Women must take on a role of reformers, reformers and at the institutions must be led by hopefully political elites that are more enlightened by the pressure of the women, the educated, the forceful women, to introduce the demands that the societies that guide all these different institutions, that they must reform. That include the catholic church. I mean it’s uh the changes of the catholic church is peanuts compared to what it should be. I mean I consider the catholic church, I served the mass for 6 years, and I’ve been a catholic and so on. I consider it quite misogynist. And so that whole framework has to change. And if you tell me yeah there are traditions and stuff when we go to different societies. Traditions are a value when they continue to assist humanity. When they are a constraint to human beings, no matter what group they are, they are of no use for the society. And that takes guts to do that. So who else?

Moderator 1: Can I ask a quick question? I was just listening to what you said and I don’t want to take it away from any of the students but maybe a cliffhanger but some students were asking about your personal career and maybe the cliffhanger would be when you stood up uh when you stood up that you as a general said I am suffering from post traumatic stress disorder. Weren’t you doing something already that you just outlined?

Romeo: Its quite uh...thank you very much for that question. I am drinking cold tea it’s, it’s uh, lunchtime here. Let me set the circumstances of it. I was realizing that I was not performing the way I should be. I was also realizing that we were taking significant walking wounded casualties. Also that the uh PTSD uh is can be a terminal injury, suicides were happening, families were being destroyed, women were being beaten, men were drinking, they were losing careers, they were in the streets. It wa- it was quite quite catastrophic. So there was a lot of stuff that was making it ugly. Uh however uh what broke the code for to turn the beyond just myself and I was a 2 star general uh, uh not a commander, field guy and all that good stuff uh was I was I was being told by the doctors and by the lawyers uh that uh we could not deliberately attribute the 11 suicides in a regiment that came to Rwanda and it was nearly after the war uh…to uh their suicides to their experience there. The traumas that they lived, the moral dilemmas that they had to suffer through, the ethical dilemmas they had to go through. And so uh they were telling me that they, these factors were not the dominant factors to why these men had committed suicide. And they wanted met to go public uh to having uh been uh in uh in uh the wars and the experience, I hadn’t gone public yet uh that I should be able to explain that the that these are not uh fundamental factors to why they committed suicide. So when I held the press conference and it was a lot of people there and so on, I told them that those 11 soldiers had killed themselves because specifically they had gone through the moral and ethical dilemmas that they could not find the solution to, that they had been traumatized beyond any of the value frameworks they had lived under and so that tipped them over the edge and destroyed themselves because of it. Well that didn’t go on well but in the same press conference I said and by the by I am one of them because I had already attempted suicide by then. And so to be able to make the point one must be personally engaged. If you are going to changed human beings, you yourself must gauge as a human being. There’s a price to it but you can still be very fruitful and still conduct a normalcy of what you want in your ambitions,  but unless the passion in your gut is so real that people can just see it in your eyes without being fanatical but by being objective and true, you will not convince until you have to engage and that’s why I consider under 30s to have established a certain passivity, btu yeah its true in Libya and Tunisia the young Arabs did move the yardsticks but they lost steam. They lost steam, they got overwhelmed by the weapons and the you know changing of regimes and so on. Um we had a strong movement of universities across the world in helping XXX and again lost steam. Uh, Uh you know 2005 I was involved there. Uh They have to sustain an activist role beyond what anything we’ve done before and use the social medias as their primary weapons system to uh to be able to coalesce in real time and build he capacity to influence.

Moderator 1: Thank you for sharing this. Now uh um I am looking at the table. XXX or XXX or XXX take your chance of, of using this as a cliffhanger to raise your question. Uh I see I see a finger first from XXX may- maybe just introduce where you are XXX. 44:06

Student 5: Yes uh good afternoon everyone or good morning depending on where are you. Um well thank you first for the opportunity for being in this very interesting um conversation uhm I am XXX. I am from Mexico and I am studying- I ma a masters student in international children’s right XXX. Um so-

Moderator 1: At Leiden University?

Student 5: Leiden University yes. And um well I hav- I don’t have question um but I have like a comment fr- fr- from the prior uh topic we were talking about raising awareness about feminism or um ge- this generation and we have to take action. I strongly agree with that its’ its very important and uh its uh very important that we uh in this uh type of uh discussion tables we, we raise those topics mm. Nevertheless I believe that we sometimes we ha- uh don’t have to forget that uh this is a XXX in many regions for Example in Latin America where I am from, uh most of the time these uh being a taking leadership in feminist its really like uh, uh choice of bravery because of sometimes uh its like a civil war there uh where if you like the feminist movement improves or, or takes us a step forward then like this society may most of the time sexist of the society not everyone but like really um can reach levels of life threatening or like uh really it takes uh takes uh a civil war I would say so its uh its uh even I think um an extra barrier of like what we already have in this part of this world for example. I think it’s the same in Rwanda or so for so for and-

Romeo: There’s so many, there’s so many societies either by tradition I’ve seen so much in Africa uh by religious relig- restrictions have subjugated women to uh the border of being slave. Uh, Uh it is its is not an atmosphere where women are treated as equal human beings, they are simply uh instruments of producing children an responding to what the male dominated philosophy of life and religion is. Uh there is no doubt that there is risk and that there needs an enormous amount of courage and so on. That’s why uh I think you’ve got to look at you numbers, you’ve got to look at who you are in the society. You’ve got to use the force multiplier that the social communications and real time instruments provide you. You don’t go at it alone. That that’s gone. We don’t we don’t need individual heroes  you know to be burned at the stake, or shot, or stuff like that. What we need is a momentum of youth and the momentum of women engagement that commits itself with an overwhelming force of change and deliberate. And that means bringing in those under 30s. Those over 30s yeah if they want to. But really its uh its 30 is a good reference point in regards to mastering the information world and also mastering the desire that you realize the country to me when I graduated was 20 years down the road because of things changing so slowly. Your future is 4-5 years down the road. By the time you graduate things have changed. And so you don’t have the time of apprentice that I had to be able to establish things and build your sort of uh, uh social presence and, and you have to face the reality of significant change and reforms every 4, 5, 6 years socially, technologically, economically and uh, uh, uh dip- and politically. And that requires your engagement now, but it can only come from the massive engagement of uh women into this belief that they will be able to change the male dominated construct of the humanity because we can talk about whole humanity, the male dominated construct of humanity and all its institutions and its and its ways of seeing how to advance humanity into the future. And and, uh, uh you know I speak to students and universities in my country here and I remember a number of occasions they come back and say well we cant do anything we’ve got no power, we’ve got no influence and we can be you know held back and and, and so on and so on. And I said that’s totally wrong. I said in our democracy, the last election lets say, uh and I am not negating that in democracies its easier that is certainty and it could be the reference point, but in uh in our country the last election there were 2.9 million votes that were not used from those between the ages of 18-28. Okay. They didn’t use em. Now if those 2.9 million young people had decided to vote, they could have changed the face of politics in this country in one election. If they got together well vote a new party to build up one party or another. So the youth of the world hold the balance of power in the democracies. You hold the balance of power in the democracies. You can start right there and build it up and use it.

Moderator 1: This is a nice cliffhanger to XXX. XXX asked a question yesterday about courage and maybe, maybe you can frame it to any way you want. Could you could you speak to what you what you wanted to bring to the table?

Student 2: yes so we had a conversation yesterday 50:53 about continuing to work for Untied nations or within politics um after coming back from uh Rwanda and working through PTSD and kind of the courage and persistence and growth that takes to be able to do that. I guess my question would be like what tools did you really use to keep yourself going to work within the politics and work for UN after Rwanda?

Romeo: Well one of the first things was first of all try to continue to work uh continue to try to find a reason for living because uh by then by the time I came out I didn’t really see the logic of living anymore. I mean how can I be in the midst of hell and I just fly from 15 minutes beyond the border and everything g else is normal. You know the people just keep on going. So the dichotomy of this was, was, was catastrophic and a shock to start with. But in regards to pur-pursing I think that one of the first things was to drive. You literally become driven by wanting to overcome uh the impact of the horrors that you and the trauma and the dilemmas you’ve been living with and actually push yourself uh to be to accomplish small things uh minor things, but evolving things. That’s, that’s one desire you say I am going to fight it. I’ve got to fight it. So that was the, the first point that came out and to do that you have to find a cause. Not an easy simple cause. Find something difficult, long term so that you don’t get disappointed by doing something, and it doesn’t work and you get disappointed and then you give you up uh Give yourself a mandate which I gave myself with child soldiers as a as a mandate. Stop the use of children as weapons of war. Then in regards to working with the po- politic world and so on um there were many, many occasions of emotional uh being emotionally overwhelmed by the futility, by the uh um the [inaudible] the child like mannerisms, by the opulence of our societies uh, by the realization that um uh they- they just didn’t’ sense that the people who were suffering were just as human as they were. That they just didn’t come like I am fighting very much for the [inaudible]. And, and, uh you know its it’s a I can’t get the people to understand that these human beings are just as human as us. They’re just the same as us except they are being massively destroyed by a government that is deliberately doing that and having seen that in the past. So in order to pursue with that it, it takes help. You wont be able to do it alone. You need formal therapy to set up some parameters to help you both psychiatrists and psychologists who don’t always get along together. Uh I had a psychol- a psychiatrist who gave me pills because of all the bad dreams I was having and then I had a psychologist who wanted the dreams in order to help me solve them. So every now and again you have some of these little problems but you need professional help. Secondly if medication is required you take it. They don’t turn us into zombies anymore. They just give us a chance to not live like this but in fact live reasonably and that’s very important. And the third element that you have to build on and this is critical between sessions and to build the resiliency is to find peer support. You got to find somebody who is willing to listen to you for 3-4 hours and not ask any stupid questions and just listen and listen and listen and cry with you sometimes and laugh with you sometimes but you need the peer support as a backup in order to help you in a crisis moment and also to sustain uh the difficult effort that it is to build at some point uh level of resilience that will permit you to do the fight. So still today I take 9 pills a day you know and I’ve had therapy for nearly 20 years uh hey but uh its worth it. And so don’t try to do it alone and families are not necessarily was the best solution. They are too emotional, too close, ask too many stupid questions, you know. Find a peer support from the work environment, from an old uncle, a really old uncle that you trust whose willing to listen without talking. That- or aunt. Those are the solutions.

Moderator 1; Thank you thank you XXX also for uh bringing that up and, and, and maybe that’s also a cliffhanger. XXX it relates to your questions that you had yesterday. If I may give you the opportunity if, if, it …yup

Student 6: Um so thank you again for being here. Um my question is around um mental health and kind of the stigma that surround it and bringing it back to I guess male dominance society if you could talk a little bit about how like stigma plays into um the idea of like PTSD and especially when it comes to moral injury and kind of how you navigate that um kind of conflict that goes on.

Romeo: yeah. Let me give you an example in the workplace uh from from XXX to show how uh the social acceptance that we have um, um nearly reinforce what you just described. Um you’re in an office with many people and there’s all of a sudden one of the ladies uh has contracted breast cancer and so the word gets out that she’s- and that she’s going to have to go into therapy and takes some time off and hopefully you know not have to be operated and uh you know hopefully be successful. So uh the office team all gets together and they have a cake and they have a little sort of going away sort of uh congratulatory you know with a few flowers saying all the best and fight it and succeed and we’re looking forward to you coming back. Then also in the same office you’ve got somebody whose been overwhelmed by the uh rate of the work, by other pressures from home, wh,o who is falls into a depressive mode, that maybe has suffered a trauma, that maybe even as far as PTSD and that person is having a hard time with keeping up. Having a hard time being able to sustain the pressures of the job and so what happens in our cases that she recognizes that oh geez you know she’s not performing not as like before and, and then wha- one day all of a sudden she doesn’t come back to work. You know she’s on sick leave. She very quietly sort of uh organized. And she’s gone for 3, 4, 5 months on sick leave. There was no party. There was no going away. There was no encouragement. There’s nothing else. This person just disappeared and then when they come back, the one whose survived the, the, the breast cancer and so on has received a nearly a hero’s welcome. You fought it, you won, congratulations, you keep on going, we’re so proud of you and all that good stuff. The one who came back because of her mental difficulties that she’s had in tr- it’s oh you’re back, oh good, and, and I am glad, and I hope you can keep up now, and we’ll, we’ll see how things work out. The mental dimension is perceived as a minor compared to a major physical injury. And so that’s why we coined in 1997 uh after I went public the term uh operational stress injury because when we called it uh uh mental problem, or mental health, uh the soldiers didn’t want anything to do with it. When we called it an operational stress injury, and it was honorable to be injured in that way, it changed totally their philosophy in regards to getting help and support and so on. An so you’ve got to frame this problematic of all these walking wounded that you have around you in as much as treating them with the respect, treating them as people who are injured, not sick, and that that injury which came from the work environment, or mixed with the family environment or whatever, that that injury is honorable and that they deserve all the care and the same concern and sense of urgency that you give to somebody who is physically injured to some who is mentally injured. And once you’ve established that, uh then you see this this stigma dissipating because it becomes acceptable, it’s honorable. It’s, it’s a norm. Somebody busted a leg, somebody’s got to take a gall bladder out. Somebody who’s got a mental difficulty that needs to be uh treated. All the same same sense of urgency. That’s the way I go about it. So anybody else?

Moderator 2: XXXX I think you’re muted sir.

Moderator 1: Oh right thank you, thank you very much alerting me to that. We’re on top of the hour. Are we still doing okay for a little overtime?

Romeo; yeah maybe another 10 minutes and I got to go to another conference.

Moderator 1: Okay. I wanted to put somebody up to the to the to the mic who doesn’t know I am going to call on his name but he’s a special person. He’s the son of two of my former classmates, when I when I went back to high school and his name is XXX. And he said dear can I, can I have the privilege of being with you. He studies um holocide and genocide studies at University of Amsterdam. He wasn’t with us yesterday but is there any question XXX that you would like to bring to the General’s awareness or any question you want to put forward?

Student 7: Uh thank you uh XXXX. Uh I think I’ve like a million questions I would be able to put forward but one that perhaps is in line with what the General has mentioned before, you have mentioned regularly in the last hour the young people, that the youth, the future that we should be able to build, uh but you can’t really build  a future without looking backwards right? And when I look at the Netherlands, there’s only a very selective-

[Unintelligible]

Student 7: someone is off mute I think but

Romeo: but yeah go ahead.

Student 7: I think in the Netherlands there is a very selective aspect of history which is which is taught in university and high school so uh when you look at Rwanda for example I think a prime example of the failure of humanity which e can learn a lot about, it’s hardly mentioned at all in Dutch high schools. So what I was wondering really if you have like a clear idea of what young people can learn from examples like Rwanda from your own experience you know on which on which we can then continue to build a better future which I think we can all agree on.

Romeo: I think you’ve you’re, you’re what a lovely question. Um and um my XXX who is with me in Rwanda uh Lieutenant Colonel in your army, just retired. XXX a magnificent man. Uh I think that what uh you’re we’ve got to bring forward in regards to societies remembering the catastrophes of the past, is the institution of our thinking that all humans are humans and that no one is more human than another. That is to say they didn’t, nobody came to Rwanda, because they were all up in Yugoslavia and uh they felt that the sub Saharan black African was less important as a human being than Yugoslavia and in so doing abandoned them completely and treated them as a lower class human being. Uh the tsunami in 2005 in Indonesia was at the same time as the XXX 4 catastrophic movement of 100s of thousands of rapes and killings and 100s of thousands and so on uh…the whole world responded to the tsunami with but nothing came to XXX 4 for human beings. And so the essence of all this is the essence of what human beings are. We are now all identifiable in humanity in real time. Its’ possible. And because of that uh we cannot continue to do a pecking order in humanity based on self interest of nations,. We also must be prepared to pay sacrifices in order to assist in advancing humanity and all of humanity and part of the sacrifices as an example has been sending in peace keepings int countries and losing them and, and taking casualties to help others. That is part of being uh, uh a member of humanity and of a particular society. Where we lose is that our political elites have demonstrated a flagrant avoidance of wanting to take whole of a new concept that came out in 2005 uh out of a norm really out of the UN, called responsibility to protect that says that its not the state that is sovereign, it’s the individual that’s sovereign and in so doing whenever massive abuses of human rights are happening, we all have a responsibility to go in and protect the innocence civilians in order to stabilize the situation. So uh until nations go beyond self interest and stop being dominated by purely self interest and risk averse uh, you are going to see people let go and forget and not put an emphasis on some of the catastrophic failures of the past that will continue to repeat themselves. So the biggest blame of all that is in the political elites of the nation that are totally, totally focused on self interest of their nation. You want proof?  Look at how they are distributing covid-19 vaccines. You want proof? There it is. Humanity is still locked down and we are I feel that we are even going to see more locking down of countries and closing of their borders into their sovereign states versus realizing that the young people of the world who can communicate globally to the world and sense what humanity is, and what human rights are and environment is in the grandest scale, they are going to disconnect with youth and it’s the youth that’s got to fight to keep those borders open. The world is not, humanity is not driven by Borders, humanity is driven by the respect of all humans being equal. And that’s my short answer.

Moderator 1: thank you [laughs] thank you so much- Romeo; And I must go I afraid because I’ve got an urgent call that just came in. Is it okay if everybody [inaudible] to just say your gratitude, briefly open your mics so that you can say a word or two to the General? No. Well if you wanted to just open your mics and say gratitude or wah, wah, uh, um acknowledgements to the General for being-This was very invigorating. It was a call to action uh, uh Romeo and I see a lot of students already nodding. It was historical to bear witness to your presence here and to give you this this revolutionary perspective on the how I would still phrase maybe feminization of the society with call to action.

Romeo: nothing, nothing like being uh being a retired general that I can talk about revolution. Its uh its uh I got to go, I wish you well and I look forward to the next 3 sessions. Bye, bye.

All: thank you

Moderator 1: for those who don’t want to sign off yet, stay on for a few minutes if you wish. The General has to sign off-

Moderator 2; I think XXXX is just trying to get back in the waiting room if you are able to-

Moderator 1: Oh there is XXXX. That’s wonderful to have her with us for a short minute. So for those who need to go who have pressing appointments or whatever we, we, we we’re okay if you sign off. Uh I could not chat with you all. That would have been helpful because my zoom version doesn’t allow me to chat with you as you were talking. Hi XXXX uh but if you want to stay on for a little bit we could chat for a little bit about how, how in the aftermath how it felt or what questions were not answered or not, not asked and uh we could give probably XXXX a little bit for an update of how it how it went uh XXX if you wanted to just just brief XXXX of what we’ve just heard and and-

Moderator 2: yup by all means. I’ve got uh well I will have hopefully about 10 minutes and-

Moderator 1: sure, yeah, yeah

Moderator 2: and then another thing at the moment as well. Um wah-

Moderator 1: let me say this for those who are signed on just- I thought it was brilliant my, my take and if you have to go and and sorry that I I was not able to address all of your questions that were asked yesterday because ther was ther were too too, too many questions to, to steer but uh okay so XXX go ahead.

Moderator 2: No wonderful I think I agree that it was brilliant. I uh it was a privilege and a pleasure not only to be in the same zoom room uh with the Gen General. I think the questions were amazing. Uh his um the focus of his, his first points I think uh related to a call to action for the youth and uh a reaffirmation fo the importance of the youth uh using some of our wonderful social media tools. I think he called it as a weapon of mo- mobilization I believe. Um as well as calling for uh um a, a real and true equality of women and men and a real push uh to include uh women and women’s way of knowing and being within the leadership ransk. Um I would I would’ve had a million questions I wanted to follow up with on that but I recognized that it wasn’t about me and I think I’d like to thank you XXXX for uh a wonderful job uh um if the uh f- facilitating this meeting.

Moderator 1; it was a pleasure. Thanks for that summary XXX. And any, any, any questions you would have now or that things that came up that you would like to bring to the table or things that came up or stirred up or thoughts that or whatever you want to share. Go ahead. XXX

Student 1: yeah it um as XXX was saying it was fantastic opportunity. One of the things, a question that was asked um nagging at the back of my mind was how do you teach them moral, morals in general? And I thought a lot about um like teaching it in a didactic concept at universities but the way that uh a revolution happens its not going through stable structures. So how do you teach or how do you create more communities such that you’re um weaponizing these perhaps more of a cost of language, but weaponizing social media o create community iunt hat way such that you can act on moral behavior. In light of covid is a fantastic example of uh using and I don’t want to say shame but in some ways shame to have people stay at home. Is that a pathway to moralism? So half baked question but I was curious to have folks wrestle with creating moral communities given the tools we have.

Moderator 1; Yeah and how we teach those. Yeah.  XXX

Student 8: as I was um I was really interested in what he was talking about when he was saying recognizing that all humans are human and how, how, um, the you- young people o f the next generation can be able to use technology, to be able to see a world without borders and use it to be able to make a difference  and I really was wondering um just like wah- what some of the challenges can be with the technology as it is so rapidly changing ll the time and how that could actually impact how we connect and how we see one another uhm it was coming to mind as he was talking about that.

Moderator 1: yup thanks. XXX

Student 2: I think one thing that came to my mind once again a very half baked question was like the concept of maintain steam. Like especially with things like covid right. So policies are so cha- slow to change, social systems are so slow to adapt or be modified that, how do you really continue to maintain steam within a group in or der to actually until you actually see change or a big enough change.

Moderator 1; say that again so how do you make change in a group?

Student 2: Yeah so how do you continue to maintain steam because he was talking a lot about like there’s a lot of initiative that do start up but they kind of end up dying off which is very true and how do you maintain those initiatives because changing policies is a very slow process.

Moderator 1yup, yup. Uh XXX.

1:16:21

Student 9: Um I am going to tie what XXX said what XXX said and I think about XXX said um it think that in the past often literature and story have been a vessel for moral teaching um but current societies definitely more based around sound bites of information and general Dallaire did bring up the convey 2012 movement and video that I am sure we all saw where that did touch everyone morally but um it did fall off rather quickly so that was kind for the new technology that XXX brought in that…It can be used to still see people as human but then as XXX said um that movement in particular did lose steam and maybe it was because of the direction or maybe its like just that social media does sensationalize things really quick so General Dallaire did say that Coney saw himself as a god after this because he was so well known by these celebrities. So yeah I don I don’t know if there’s like a lasting um or yeah you think of great literature and works of the past and they’re still great literature being written that always seems to be a comment of current society and what societies thinking about and those artists are often a little bit quicker to see some of um the issues that we’re currently facing um but yeah now I, I just have dot dot dot I don’t really know where to do with that um but if anyone else has a comment and I also uh maybe not to talk for too long but I did have a question as well for general dallier because he was talking about implementing a feminine ethos within STEM and other um male dominated uh institutions and I was uh my question was going to be around healthcare and a lot for healthcare-

Moderator 1; yes that’s what you said yesterday right? The healthcare workers

Student 9: yes um and a lot of health care organizations have really tried to develop an ethos and it usually is um informed by somewhat of a feminine perspective of care um but then like during COVId-19 and not sure COVID-19, I was actually talking to XXX, Dr. XXX who is on the call right now, yesterday and how Covid has actually just highlighted a lack of resources that are causing moral dilemmas for healthcare workers so even with some feminine ethos there’s’ still um just a lack of resources and things going on that are causing moral distress in people.

Moderator 1: is that a question for XXX you wanted to ask that now to comment on? Ask her to comment on?

Student 9: uh I suppose I was just pulling in um some of her own current uh research and current exploration into the topic of moral injury within healthcare workers and I um was going to get General Dallaire to comment on that today if there was time permitting.

Moderator 1: Time, yeah yeah. XXX are you with us? I can see you but did you hear the question of XXX if you wanted to speak to that?

Student 10: Sure sorry I did yes. Um yeah I think it’s a really interesting point and I think ultimately what I’ve heard in the last few comments-questions and I think this is always really at the crux of the issue is how do we create lasting and effective change? And I think ultimately part of that then is what are we striving for and I mean in the question Dallaire, uh you know General Dallaire posed to us in terms of all humans being human I think underneath that is the question of you know what does it mean to be human and what do we need to thrive. And I think some of the time where you see you know sort of um movements sort of come and go is because I think they can often go because they don’t necessarily address those those r- issues or they’re struggling to continue sustainable in light of the challenge so I think we’ve all seen sort of NGOs sort of pop up and sort of save the world but then when they actually get into the issues and I think this is why I think part of it is really helping people to empower themselves um to actually bring those issues forward. So to tie that to what XXX and I were talking about last night with COVID and, and the moral injury I think part of it is COVID hasn’t necessarily created moral injury in healthcare workers. COVID has allowed us to understand and actually see the full scope of the moral, ethical um dilemmas, and challenges and injuries that these people have continuously face but up to this point been very minimized and sort of downplayed as being sort of secondary to other challenges. And so I think it’s a really wonderful opportunity when we think about moral injury in COVID to actually be able to hear I hope when we come out of COVDI the struggle of healthcare workers and to actually engage them in a meaningful dialogue. Everything from what do you practically need in terms of resources but then what do you also need as to be fully functioning, viable, human based health care workers. What, What else do you need? Because we know you have been struggling especially if you’re in Canada for a long time but we’ve just kept kind of papering over it and come up with these new crazy ideas and we try it. Um so I think covid in some ways has allowed us to see the bubbling up of all these pieces. So I think it is uh it is hard because ultimately it the fundamental issues I think often stem from our, our indifference right? Its uh, we, we got very emotionally involved and I think um my criticism of social media is that we tend to do that very quickly and then when it comes to the next thing then we move on after that right? And so the same thing can happen with covid. We’re all very interested in covid and we’re all very interested in the consequences of covid but what happens when covid is over? Do we then stop and say okay we just did 18 months and we need to seriously look at and examine our health care system. I am not sure we’re going to do that. I think we’re just going to move onto the next thing. And I think that’s often where those moral injuries then lay because we just repeat the same cycle again.

Moderator 1: Thanks for chiming in XXX. Now we’re going to the bottom of the hour probably then we should stop uh because that’s about 6 minutes. Anything er that have been left untouched or some? Any of you? XXX or um if there have not been any remaining issues then uh…no really? XXX are we missing out on anything that you wanted to say?

Student 11: No I am just generally grateful for the opportunity. I think it was really refreshing. I really appreciated the beginning like to talk about the need for more women in power roles and I thought that was really an interesting perspective coming from somebody who has a history in the military where it is so male dominated so I thought that was really refreshing perspective but no questions just kind of in awe to be able to be a part of that.

Moderator 1: no just want to hear your voice [laughs]. XXX anything you wanted to share?

Student 12: Kind of similar to XXX. I am just kind of soaking it all in and mulling things in my head right now.

Moderator 1: okay, okay. Who didn’t we hear yet? Have we heard everybody’s voice? XXX we heard you too right? So I think just wanted to say that this was a one time opportunity. There is no rehearsal or second opportunity. This was a one time thing. Its over now and then we’ll continue through our courses. Its been historical and revolutionary perhaps in a way. I feel that this is in a way historical because this was a call to action. What I just heard the General say this is a call to action I felt and uh I am not uh I am not under 30 uh we learned yesterday that most of you are under 30so I feel this is a call to action for you and most of you are feminine but that doesn’t it doesn’t need to be XXX your sex or so. You could be you could be invigorated because you’re male here and see if you can uh its not only for gender specific call that he made. Um just just a thought that comes up and we’ve been talking about this Moderator 3 as well when we were talking bout tiers for PTSD and we’re talking about moral injury and we’re talking about novel opportunities for treatment. I think there’s also a different quality to how you repair and how you XXX into things like shame and guilt and forgiveness. Forgiveness may be a thing and an opportunity that that needs another quality to be remedied or, or cured in a way and id like to speak more to him, how he, how he incorporates maybe religion or, or things that that come from a different disciplinary than the than the pills, and the psychotherapy there, but that could be for another opportunity. Maybe what i’d like to say in in closing um what I feel you’re all students is uh I am not your supervisor but just read about, write about this uh I think that would do service to what the General said. Incorporate his thoughts to whatever you write and take the liberty of uh of expressing your ideas with what he just shared and if it fits and if it makes sense then it just justice to his moral courage call I feel. And talk to XXXX and, and to XXXXX about this and XXX. They are at your hand. I was delighted with you question XXX. We don’t know each other [laughs]. It was brilliant. XXX called out to me maybe a couple of months ago and really he is a son of a classmate, 2 classmates who were a couple at those years. This is 20- how old are you XXX? You are?

Student 7: 22.

Moderator 1: 22 so this must have been over 25 years ago and they got married and they had a son and XXX reached out to me and said hey I am the son of my two old classmates and I am now studying genocides studies and I heard about Dallaire. Can I just join in? So I think you uh I think you raised a brilliant question. How do we teach this uh? Right uh, uh you are not born in 1993 yet, most of us were no, you were not born but how do how do you learn from this? We have in the Netherlands XXX, there was all the action that was XXX in Yugoslavia. And that our black page in Dutch history. But we could go on and on. We’re almost at the bottom of the hour. Moderator 3 how does this feel when you hear these conversations because I haven’t heard you yet?

Moderator 3: it’s exciting I um, um just regretting I missed the rich conversation. Um I just want to get a sense from everybody if it was valuable for you on a scale from 0 to 10. If 0 was-

Moderator 1: ah the psychomet-

Moderator 3: was this valuable? Okay awesome

Moderator 1: [laughs]

Moderator 3: would you encourage other students? We have 3 other opportunities of these?

Moderator 1: yeah we do

Moderator 3: okay. Would you also be open to writing up a- this is an optional, this is an open gift for you to experience this. We would be grateful to hear some reflections from you if you feel like sharing and we might even compile those as part of the book that we’re planning on building together. Just some reflections from each of you that we would put in a book uh all of the lectures that are going to be happening in the fall, the speakers that are going to be coming. We’re taking what they’re speaking on and compiling it into a book and giving it to the General as a keepsake that could also be available um for others as something that can carry on in time and so if you would be so kind um and interested in writing something that could be that could go into a book. It might, we might have to edit it a little bit, I don’t know but if you’re’ interested in doing that I put XXXXX’s email and also my email in the chat um so if you could send it to us we would be happy to do that. Um sorry you have your hand up XXX?

Student 3: yeah I am wondering if we can access a copy of this recording for our own reflections and note and stuff.

Moderator 3: Um we can give you access to it. My only comment is this, that we’re committed to all of you and the General that this is a closed concertation so if we share the video with you, it cannot go anywhere outside of this group um so that’s my only caveat um it can’t show up on social media, it can’t show up anywhere else. It would be for your own purposes so if you people are committed to doing that and we have confidence that that’s the case, we’d be able to share the link with everybody. How about I what don’t XXXXX and I haven’t talked about that or XXX or XXX. So maybe what we can do is come up with even just an agreement from everybody that were not, no one’s going to share it because this was really a closed door conversation um and then we’ll uh, if, if people send back the consent form, we’ll shar the link with you. Maybe we can do it that work? Would that does that sound reasonable? Yeah. XXX, XXX does that sound reasonable for you? XXX?

Student 10: Yeah I think so absolutely

Moderator 3: okay so we’ll, we’ll prepare that. I am grateful to hear that there is such a stimulating conversation um I hope that it was a rewarding one and an memorable one and we’ll look forward to, for those of you who feel comfortable, sharing some of your afterthoughts and reflections and seeing if we can incorporate that.

Moderator 1: please do, please do.

Moderator 3: we also-one more thought. We also are maybe looking for some people from the students who are engaging. So there’s 4 sessions. We will be looking for some people to actually go through all these and able to do an analysis, a thematic analysis of what were the key what were the key topics that the General and that everybody talked about. Um and what were the what were the nuggets that he that he gave. If that’s of interest to you, let again, let XXXX and I know if you might be interested in doing that and supporting some of that. That would be a research project that we would be kind of putting forward so if you’re interested just note that too. Okay. Thank you

Moderator 1; thank you. Great closing comments. So its now uh 6:30 local, central European time, its about noon, no, no, no, no, no 10 o clock your time right?  Right? 10 o clock your time. So enjoy the rest of the day and um thank you Moderator 3, thank you all for being here.

All: thank you

**May 5, 2021 Session 1:55 duration**

Moderator 1: We now have also XXXX who was with us on one of the previous student engagements with the General. Before we get started we would like to record this session because it is important for us to revisit if we wanted to do that because a lot of it is going to be too fast to capture. Is anybody objecting to this being recorded? If not so XXXX would you like to… the recording isn’t going to go out to anyone who is not here at the table. Dear General, Dear Romeo, these are the students, part of them are from the Netherlands, part of them are from Canada, the University of Leiden and the University of Alberta. We are happy that you are with us and may I give you the, how do you say, the floor, It’s a little odd to say that. May I give you the word I should say..

Romeo: Thank you. The waves.. the ether of the… thank you professor again and professor XXXX for keeping this colloquium sort of going. Welcome to the you the students who have been quite diligent in sending me some absolutely wonderful questions. I’m very happy to receive them because I feel they are questions that insight of worthiness do give the opportunity for discussion. So I’m going to use a number of those to speak to you on the next four hours, though I’ve been limited to three or less than that. But I do hope that these opportunities will open up other ventures for you. I think the first one, the area that I would like to touch upon was on the moral injury and responsibility of command. This will lead to many other questions that you may have on ptsd and the impacts of these complex and ambiguous missions on the troops and of course the nature of conflict for which we find ourselves. So if I touch on those things I touch and I hope I touch on a few of them, and do make sure I leave some time near the end for specific questions. I don’t want to go off eternally. I think the first one is that regarding how one finds oneself in ethical and moral dilemmas in command of missions that are of the complexity and ambiguity of our time. We find that mandates that have been written and produced are not based on some very sophisticated doctrinal assessment, political science results but are often so politically over ridden by self interests that and resource availability is that what we end up with in the end is in the field a mandate that may still be very demanding but the resources are not their to accomplish it. And that creates an enormous dilemma on whether or not you can pursue and achieve the mission that the people who have been fighting the people who are looking for peace are actually facing and they can very rapidly turn against you if in fact there coming as an asset to peace, only to find that you do have the capabilities of influencing of the evolution of the peace process, the implementation of it, the continuum of reconciliation and ultimately in fact bring peace to the nation. So one of the great fallacies of our post cold war era has been the fact that we really had no capabilities of meeting the imploding nations and failing states and mass atrocities that were ongoing. Exploded by the world disorder that George Bush coined the world order after the cold war but in fact we ended up with a world disorder where many nations who were artificially created through the colonial era and so on sort of internally imploded and in so doing civil wars and mass atrocities became the order of the day and we from the security side had a little to no capability in that measure. Sure we had millions of troops being in central Europe being able to defend the sort of Warsaw pact against the NATO construct but that Eurocentric capability had no offshoots that could meet the very demanding milestones of these conflicts because mass atrocities were going very rapidly. The number of places of imploding nations and ultimately also realize what use of force if necessary, should be in the rules of engagement and where does in fact the use of force fit in to a peace process. If Chapter 6 there to observe, how do you protect civilians? So the 90s have been an era of stumbling in and ad hocery and often crisis management that have created enormous traumas and difficulties in the field for those who have tried to implement them. Created enormous amount of frustration by nations who were throwing some troops and some money in to these processes because they didn’t see the results coming out and they were not going any further than what there self interest would want to be met. So so many of the missions were understaffed, under equipped, under scaled and particularly under trained to handle this new era of conflict and imagine trying to even talk about preventing these things from happening as we were trying to even handle how to resolve the conflicts that had been launched off. So, in the interesting question of facing a mandate that couldn’t be met or wasn’t being or wasn’t being supported what do you as the commander do? The questions was how do you sustain your troops under such conditions. So, the first thing was the example that I would like to offer in regards to the situation generating in Rwanda politically and the security situation getting more evident day by day. So at one point I was able to [inaudible] to get some intelligence information that the government was planning to conduct mass atrocities that they were moving weapons and by doing all that they were breaking all the elements of the peace agreement. So I informed my headquarters the UN, DPQO, the secretariat that I was going to conduct some offense operations to not only dismantle the ability of these forces who were pretty well in the extreme element of the one side from being able to conduct these operations but in fact to break their momentum and make them ineffective. So I got the fastest response I ever had from the UN saying you are not allowed to do that, it's not in your mandate, you are a chapter 6 mission, and so you can not conduct any operations on your own you must then negotiate with the two parties to find a resolution to that. In my opinion that was a guarantee of catastrophic failure because there was an unwillingness in fact to negotiate politically it was already stalemated. So when you're faced with a question like that and you know that those weapons might be used against your own troops let alone the people you are trying to protect you got two decisions, one I abandon the mission that is to say I resign on principle. You don’t understand what is going on, we have to do it. I'm out of here and replace me because I am not going to continue to command a mission like that. That, really, honestly for a Canadian general in the middle of Africa for a mission that had very little profile, would have been about 15 seconds on CNN so the other option however and most appropriate one is to remain with your force and to argue an attrite the authorities above you to ultimately see your way because leaving that mission when those troops were still loyal to you was essentially abandoning them in the face of the enemy and by so doing that was the most cowardly process to do. Using on principle it sounds nice but in reality when you have the troops with you and their impacts and their lives in your hands that’s not the aim the aim is to achieve the mission and so doing I continued to fight with the UN to ultimately I did win but by the time we did too much of the forces of the extremists had been able to organize and equip. So we weren’t successful but it was significant that they had to feel that the commander was in relief of what he was doing and that ultimately the higher headquarters had to change. The other side of that is when you get you to abandoned your mission actually not you wanting to do it but… Boutris Ghali the secretary general when he decided that through the information that he received from the Belgians in particular and a couple other sources that the emission was going to be over run that the few troops that I had left were going to be simply overrun because we were protecting so many of the tootsies at the time and number of senior tootsies and he said that the world could not handle 450 more blue berets being killed, could handle 10000 runs being a slaughtered a day but. So I refused that order and in so doing this gets to the question that was asked to me, then what, I mean if you refuse a legal order then you become a rogue commander and as a rogue commander what does that mean for your troops and how can you continue your mission. So I took the decision to refuse the order to leave because I considered although legal it was immoral because by then I had 32000+ of Rwandans under my protection, that if we pulled out they all would have been slaughtered immediately, we had already seen by example a country doing and so we and I particularly we are staying and will disobey a legal order because I can not consider it to be morally correct so that’s fine for me. But then I got to turn to my soldiers who now have a rogue commander. What does the rogue commander do? They by law should not be following any more of my orders so on an ethical basis with a legal position I had to go to them and explain what I had done and ask them to volunteer to remain with me to conduct the mission and in so doing they individually volunteered to remain under a commander who was going against orders and in so doing continued to protect those 32000 people and ultimately helped in the process of bringing peace and stability. So that’s the first one in regard to dilemmas of ethical moral legal that you find yourself in these complex and ambiguous missions.

Moderator 1: Dear Romeo, I am hesitant to interfere here because you are making a series of points here. Is it ok to address one question that comes from a student by asking a student to address a question already now, or do you want to make a few points before you go there.

Romeo: No I just, one last point if I may.

Moderator 1: Sure

Romeo: The experience of Rwanda and moral traumas and debates created in me the injury of what we call operational stress injury or ptsd. Be it through command decisions, be through facing child soldiers and the use of force, more and more the moral injury compared to the trauma injury is so much more profound and all the more so by the lack of spiritual depth of individuals today to handle such a high and intense dimension of your being its to say going against fundamental premise of what you consider the right thing to do. So until we made ptsd an honorable injury that anyone could fall victim to by calling it an operational stress injury the soldiers were refusing to one get help and two those who weren’t injured were chastising those who were injured and so that broke the code to finally get help, to help troops with ptsd. So over to you.

Moderator 1: Wonderful. the cliffhanger, and I was hesitant, thank you so much, I was hesitant last time to bring this to your attention, we have with us … and I are both Dutch officers here on this call, he is also an officer in the Dutch army. He would like to bring something to your attention. Maybe … could you formulate briefly your question that you would address the general.

Student 12: Yes thank you. General Dallaire, it is a great honour to have you here today. So almost exactly a year after the atrocities committed in Rwanda, the Dutch [inaudible] he faced a similar situation in the Balkans in July 1995 Serbian troops were surrendering the Dutch contingents in [inaudible] who were protecting some 20-25000 Bosnian refugees. 8000 of them didn’t survive so as you [inaudible] also requested support from the UN and this country and the support never came. So later in his book he says he felt he was forced to fight with his hands on his back, so I was wondering in your opinion what comparisons can we draw from these two events and what would you say to future commanders facing a similar situation.

Moderator 1: Thank you.

Romeo: Is a horrific scenario of the era of the 90s when we were getting mandates and no capabilities of implementing them both from the political restrictions let alone military imposition of rules of engagement or even willingness to engage. That situation and we’ve had a few of those, that commander who was put in a scenario that he literally could not succeed in his mission unless the NATO/UN forces were prepared to use air power to overwhelm the forces that were there because he did not have the troops did not have the mandate and did not have the capability of sustaining it so he was caught also with the problem that if he became active that is to say if he open fired if he commenced also to conduct the operation then he and that small force that he had which was insignificant size force when you look at what they were facing he then would be considered to be another combatant another belligerent he would have lost totally the neutrality position that he had and it would be open season on wiping him out. So he realized that if he did take offensive action there  that his whole force would have been eliminated and that ultimately the destruction of those civilians would have continued. So there is this instinctive professional reaction that commanders over all the years of experience gather in them. As a point of discussion of realizing do I or do I not commit to this operation in order to achieve my mission. When I ordered my forces to that I had refused to withdraw, we were in a compound and had most of the senior tootsies that the extremists were wanting to get at and two battalions were deploying to take out my whole headquarters. There was no way for us to get out as was the case in [inaudible] and so the only option that was left was that if they were going to come and attack the headquarters we would fight to the end. But in that circumstance at that point in the scenario that I was living was one of eliminating totally the mission and the ability to conduct future operations vs. a battalion in a small pocket but in a specific sort of arena. So yes a complex scenario. I think that he was mistreated by the Dutch government in its assessment of what he did because the political side of the house, although it did pay a price, never came close to paying the price that he had with his troops at that point.

Moderator 1: The government stepped down after the mission as sort of an apology.

Romeo: yes but I mean the politicians still walked away but his whole life was destroyed.

Moderator 1: Yeah, and he has a different, I won’t go on asking questions, but he has a different leadership position than you have had in the aftermath of the mission. He chose another role.

Romeo: Oh I see.

Moderator 1: Maybe as a cliffhange XXXX can we use your story that you shared briefly with us Monday. I’m not sure if this is a good cliffhanger to ask your question General. I’m not going to assign all names because we won’t have enough time to ask all questions, but XXXX you had a very touching also experience with a question embedded in it. Is it okay if you address it now.

Student 10: yes can you hear me alright?

Romeo: Yes.

Student 10: Fantastic. Hello general, it's lovely to speak to you today. XXXX I remember that I addressed a few different things can you remind the….

Moderator 1: The last… that’s why we need three hours, you had so many questions, sort of the last one that you were on the leadership arm and because you were young, you were born when the genocide was taking place and you were a child actually living in some of these countries there. Those are the parts that I would like to address the general.

Student10: I’m still not sure but I will tell you where my interest lies in this. I was, I think I was fourteen then so I was old enough to understand this was going on and I was in South Africa at the time. There was this, it was being broadcast on CNN and I remember asking my parents why aren’t they doing anything? Why is this allowed to go ahead? My father was a diplomat so he tried to explain to me as best he could that these are very very complicated situations in that the UN and if I remember correctly and president [inaudible] allowed the UN to be there under very strict and special circumstances and your hands were tied behind your back in a sense already. I suppose my issue is around the UN and what we could do to make it a more effective organization because that was the point, because this stage it seems like it’s, the UNs only power is if the members actually cooperate and we have a situation now where we have genocides going on now like the Uyghur Muslims in China and so China has enormous power there and they can you know veto things and so perhaps to talk about cause from my perspective the UN just feels like its not a particularly effective organization in dealing with these types of crimes against humanity.

Romeo: Well in fact that’s why we’ve seen an evolution in peacekeeping peacemaking, humanitarian interventions and now were up to our ears in stabilization missions that are not demonstrating their effectiveness either in places like Mali, Central African republic, as you well know. Remember at the same time we were going through that in South Africa, you were also electing Nelson Mandela, so I tell you the world had far more interest in looking at that positive than it did actually want to watch a catastrophic failure going on in a country where for the longest time people were calling it tribalism and so It was nearly perceived by the evolved world, the north, as simply an actual event in Africa because we had created these artificial countries the tribes were being at each others throats at different locations and we were just facing Yugoslavia at the time, Cambodia was going on, so no there was absolutely no value in there. So what dominated all of that, not that [inaudible] was creating problems for me, my biggest problems was that the international community did not want to support the UN. There’s a difference between the UN taking decisions with the international community not giving the Un the tools to take the decisions. So as an example the security council even though they answered to their capitals individually may come up with a mandate that they articulate if the countries do not want to provide the resources, which at the time the Americans were not paying their dues and do not want to providing the troops or capabilities, you can write whatever mandate you want but you can not implement it in the field. That’s what ultimately happened to us, is that we got a mandate on the [inaudible] there was no money and nobody was really interested in coming. Why? Why all that? Your dads a great diplomat so I’m not even going to debate that dimension of it apart from the fact that what dominates still today even after the Brahimi report in 1999 that brought in some tactical reforms, even after responsibility to protect in 2005 that said that if there’s massive abuse of human rights that we are all responsible for intervening, the world, and we all signed up to it in 2005 and its rarely if at all been used, what we do not have and what still dominates is self interest. National self interest dominates and when national self interest dominates then you get questions like do we want to take casualties in doing something like that, will we simply make the situation worse, do we really know what’s going on there and what’s in it for us, there’s no natural resources or anything. So until the political elites of the world consider all human human and equal, be it in [inaudible] be it In Myanmar, whatever circumstance, unless we consider all humans human, we will not get a implementation of the UNs ability to mobilize a response that is proactive not in fact even post and let alone being able to stop the situation. I give you an example, when I was at Harvard in 2005 I argued that we had to put 40000 troops into Darfur, because that’s what they needed to bring back the African base to there lands and stop the killing and the raping and the murdering that was going on there [inaudible]. And I was nearly laughed out of the room. And I said how can you laugh me out of the room, there are 100s of thousands of human beings that are being massively abuse in their human rights and slaughtered and god knows what else by youth militias paid by the Sudanese government called the [inaudible], and I said just a few years ago we had nearly 2 million troops in Europe protecting ourselves, where’d they all go? And by the by, what about Yugoslavia where we are pouring 60000 troops, why can’t we put 40000 into Darfur. So until we make the full realization that every conflict on earth will have an impact on us it will, it will be through pandemics, big refugee camps and so on, massive movements of populations that will destabilize countries, rage that will turn into extremism and outright terrorism out of those regions. Even the {inaudible] being engaged and caught in the midst of all that. That will destabilize in our own countries until we realize that there is no conflict on earth that doesn’t engage us if we believe in human rights and if we believe if all humans are human and if we really want to protect our own people and the future, till we believe that we will continue to see these half hearted and very eloquent means of abstaining. And I’m afraid that’s what you are facing.

Student 10: Thank you very much, I think I would like to ask one little follow up if that’s alright?

Moderator 1: And before you do so, I will prompt XXXX, we got so many subjects what is the next one we should focus on. We have already lined up, the general has addressed some of them, think along our lines… go ahead.

Student 10: The biggest thing though, going in as the UN and we need to interfere when these things are happening, intervene rather, what many countries say you are interfering in our internal political affairs. So that’s the thing that always makes it so tricky and I wonder how one goes… Thank you for your time.

Romeo: I spoke at the West Australia center where we signed that famous Australia document over 300 years ago and argued that sovereignty is no more an absolute. When we created responsibility to protect, we made the individual sovereign not the state and so sovereignty is no longer an absolute if you believe in what you signed up to, and every country did sign up for responsibility to protect. So that is the answer to the future, but we’ve seen that there is little to no statesmanship or risk taking by nations, even middle powers that could go in without the big boots of big powers and be more acceptable and less intrusive with the UN under chapter 8 even, reinforcing a regional power like the African union and just reinforce them. Until we see the nations accepting that in fact their commitment to humanity is beyond their borders and that they ultimately have a responsibility to all human beings not only their own and be prepared to pay a price even in blood, we will continue to waiver. And [inaudible] nearly 100 reforms that he tried to get approved in 2005 there were vetoed months before by the Americans who put them, who put him in the job there to bring the reforms, and the veto was done by the ambassador at the time who was the same ambassador who became the national security advisor to Donald Trump. When the world power is abusing its power then middle powers should come to floor and that’s where I feel the middle powers are failing and to be held accountable in history as not being the alternative to major power intervention.

Moderator 1: XXXXX, do you want to, yeah go ahead.

Moderator 3: I think Romeo Dallaire just dealt with one of the follow up questions I was going to pose to him and that’s coming from some of the students as well. You know the dilemma of having to deal with Chapter6 and chapter 7 of the UN charter, I think its important for the students who many of you were either not born during the Rwanda genocide or were little kids at the time to realize that this is always been a tension between the need to use peaceful measures to deal with these national conflicts and the need to use more coercive measures. So that tension between chapter 6 and chapter 7 played itself out during that time at the UN and also in Romeo Dallaire just mention chapter 8 and I think it's important to expand on what chapter 8 is all about. Within the UN system there is sort of this subsidiary principle that the UN will not be able to do everything at all times. Some times you will have to depend on regional organizations to support the work of global governance and particularly conflicts. Therefore chapter 8 allows for that possibility to use regional agency to address conflict. What is true, that the UN security council is kind of strapped with this veto principle of the 5 members. There are ways of getting around that by utilizing regional powers and regional agencies to address some of those conflicts. So I think this is one of the questions we have for you General Dallaire, how can we bypass the US system by utilizing regional organizations and regional agencies to address these problems that are sort of [inaudible] to the international community.

Romeo: well I think, it’s a wonderful the way you explain it, because remember chapter 6 was there because people want peace and you're there to observe it. Chapter 7 really came in with the 90s in reality, except for Korea of course previously, because we finally started to realize that we have to protect the civilians who are being massively abused. That’s the only way we are allowed to use it, even with chapter 6 I was not allowed to protect the civilians. And so the protection the civilians and the protection of women and children and their engagement in conflict has given us the ability to use 7. However, there’s so many restrictions often it makes it very difficult as we see, so Sudan very difficult to apply. In regards to chapter 8, you opened the door to something that I've been arguing that we can operate and create a coalition of leading middle powers in the world. Who have capabilities enough although not enough strategic lift or strategic intelligence to garner through their coalition a power that can influence significantly in conflicts and even get in there in a prevent mode where the UN sometimes cant because its dominated by the pyramid of 5 or even the big powers themselves can’t get in. So I truly believe that the middle powers have been running on the coat tails of the big powers and as the big powers make mistakes and the middle powers join in these little coalitions and essentially pay the same price the big powers do, vs bringing in new and cohesive capabilities of middle powers that can have significant influence if we were able to find that leadership. Political leadership, where is that statesmen that we need now? Ultimately I do believe that conflict prevention will come from an engagement and from involvement and particularly from the presence of women in a equal as men with youth reinforcing that argument. Being there right now we are in a male dominated philosophical framework of international affairs of all our disciplines, it's all male focused. The women have got to crack that thing open and introduce a whole new generation of capabilities that can make it not feminist, sometimes people use it as a pejorative word, but far more astute from what the women can provide. Only then we will break the code on this male dominated world and this sort of historic way of trying to find solutions in an era where the whole situation has changed.

Moderator 1: you had a call to action last time you spoke about that.

Moderator 3: yeah and that is a very important point.

Moderator 1: Its good for the students to be aware because we are representative of a older generation but the students are really the embodiment of that younger generation that the general is addressing now.

Romeo: yeah and I would argue that the younger generation is a generation without borders. They are already global. They communicating real time with anybody in the world, they have a global capability they see the world as a little fragile ball. So when you talk about climate change to them, you can talk about world climate change, you don’t talk to them about an oil spill or some garbage some where, you talk to them about the world. You can talk to them about nuclear weapons and the absolute stupidity of that capability of the bug wash movement that still exists. They can grasp that, you can talk about humanity, you can talk about human rights, because they can see all of a humanity and can communicate with them. So I am looking at that generation, without borders, that is most of them under 25, 30 who are far more integrated male female and sensitive to youths with the communications revolution to change the face of how things are done in the international sphere.

Moderator 1: So General, we hear a person with passion and talking to this group of students like you did last with passion. And one of the questions that came up is in the midst of the aftermath of what you’ve been through where you suffered from ptsd, where you destigmatize ptsd, and you lived through it. All of the students were intrigued about how you do that? Where do you get your passion? Where do you get your motivation? And the inside, specifically the passion to talk to the younger generation. How do you do that? What does it have to take in you to be able to do that?

Romeo: It comes, as I’ve tried to respond to that in the past. I’ve come to the following answer, at the height of the genocides where the world told me that there’s no cavalry coming, we were not going to get reinforcements, that they were going to let the slaughter continue, that there was no value in any country intervening, journalist were on the ground with me. They were the only weapon I had to get the word out. One journalist came to me at one point, and I had taken casualties by then and we were moving up to 50000-60000 people along the roads watching them die and sick and so on, and this journalist came to me and said why are you staying here? The two armies don’t want to stop fighting, the slaughtering is ongoing, the political impasse is still there. Why are you staying? I said spontaneously, if I can find and protect one Rwandan victim, to be the witness to the world of what happened here, it is worth whatever casualties it will take from me to do that. From that argument I said there is no giving up of this, there is no end to this because humanity needs to learn its lessons, needs to be reminded and so my enthusiasm comes from the fact that I’m never going to let the Rwandan genocide die and as so doing I will pay that price of continuing to fight for it. And my wife has joined me extensively in doing that too.

Moderator 1: While we’re talking maybe if that’s ok and there were also questions if I may direct this too, you’ve been in a lot of programs, maybe I’m not using the right word, therapy to pass through and grow. There were some, what programs helped you in order to deal after you returned and you discovered you had that operational stress injury that you suffered from? What programs helped you to grow despite the incredible burden that this had on you?

Romeo: In 97 three years afterwards, I went public with my ptsd because I was being told I was a two star general and I was to run a press conference in which we had taken 11 casualties in a regiment in a mission due to suicide. I was told it wasn’t just the missions that were creating these traumas that were pushing the suicide, it was all their antecedent way of life. I felt absolutely horrified at that, so I went to the press conference and instead of telling the press as the government wanted me to say, these were not suicide due to the missions, I said they were suicides directly due to the missions. And that we had to take the decisions to bring operational stress injuries in to an honorable injury to be treated honorably and with the same urgency with physical injuries. And so that was my launching and then I went into twenty years of therapy. I mean truly where I had four suicide attempts that I’ve been inept at, I’ve still on pills now. I taken therapy during all those years, I tried to work myself to death to literally kill myself at work. I felt that was an honorable way of doing it. I will work day and night and kill myself at my desk. So ultimately you know what saved me? I discovered love. I’ve finally found a higher calling in life. An actual loving of another human being and that love between my wife and I have now and myself was an element of backdrop to which all this other stuff could be adjusted. But without that and that answer came when I was giving a conference in Kansas city to medical clinicians at a veterans hospital and it came out again spontaneously. I said the only thing that I’ve seen now is love now, and without that it will be very difficult not only to sustain the hurt of this injury but in fact to survive it.

Moderator 1: Maybe this is a cliffhanger if I may XXXX I know what question you would like to ask. It was in a paper I don’t know if you’ve seen the New York times, I don’t know if it was last or day before yesterday, it was a breakthrough on a new medication on mdma for the treatment of ptsd… you may want to speak to that, you had a question about this, one of the emerging therapies nowadays that young people learn about.

Student 3: I’m a medical student at Leiden university, performing a literature study on mdma. It is a new treatment option for ptsd and I was wondering, mdma can help with the guilt and the emotions of the ptsd. I was wondering if you had any insight about these therapies, if you ever heard from them and if you think these can help in the treatment.

Romeo: I think that there’s two dimensions to the ptsd injury, there is the trauma experience, an ambush, accident, something traumatic scenario that has happened. And it can be repetitive and of course difficult all the more so. I think that there are a whole raft of innovative ideas and how to handle a traumatic experiences that will help you, if not overcome them, be able to manage them be able to control them and that can be done through therapy of course these different therapies, through medication I got no problem with that I do it. Those I have not spent much time looking into, in fact never even studied them to be quite honest. Because what I discovered was the injury that I had and some are now differentiate between moral injury, moral impact, is that the essence of what I was had been attacked and was under enormous pressure. Everything from the guilt feeling, from seeing things that beyond any imaginable description of every reference that your society has implicated in you, your values, and even spiritually, either or your religion or just your spiritual beliefs of what can actually happen between human beings, that side of the house I have not seen much, enough work. I know there’s research ongoing and my team is doing a lot of research in regards to moral injury when you're facing child soldiers. How many children can you kill before it starts to affect you, even though it is your mission. Even though you’re protecting other people. So your question is most pertinent. There are innovative solutions still evolving and I think they must continue to evolve to prevent as much as we can the extent of the injury, the traumas. I do believe however that moral injury, the realm of moral injury is still at the beginning of trying to comprehend how you can bring a human soul, a human being, a human mind back to a level of reasonableness without it being continuously vulnerable to sound, noise whatever that can, or smells and so on that can trigger it back to some catastrophic failures.

Moderator 1: You are the person that touched me when you spoke in Shaking hands with the devil, where you said I can not stand the loudness of silence. How is that now twenty years later? How do you reflect on that? it touched me when you said it, we’ve been revisiting that and students have seen that documentary. How is that now for you?

Romeo: The only reason I can now sleep with no, I can actually go to a bedroom to sleep with no light no sound nothing, is because my wife is with me. There is a deep deep deep link between human beings that can be enormous saviour, because without that I was not going that way, in fact I was still going towards self destruction and yes my doctors had given me at the time, less than two years to live because I was literally working myself to death because I couldn’t handle the night. I could not handle the silence. That depth of that problem was not being resolved by the therapy or the medication it just lingered on. My guilt of having failed my mission of so many people, so many spirits haunting me and so on that started to wane. I was starting to get a better grasp of, a bit more objectivity to that. But not that depth of darkness and silence that opened the door to the whole spectrum of what human beings have done to others and how the rage in those who were slaughtered still exists in the universe.

Moderator 1: I am so grateful that you are able to share that with a group with us and maybe speak to XXXX who is the expert in moral injury. Maybe you want to comment or ask or reflect on this, XXXX?

Moderator 2: Thank you XXXXX, General Dallaire it is so potent for you to give voice and give language to the depth of experience and depth of impact that the events and experiences of your leadership have left you with and the ways things have impacted you and the importance of that spiritual dimension and that potency of how that affects your very core beingness and that these things reside and continue to linger with us even after the ptsd layer has been dealt with. I think that’s the thing that your touching, that there’s something more about humanity about the essence of who you are as a person and how a fracture and the burden and weight of so many is what you’ve been bearing and carrying and how important that reattachment and connection with yourself is and with others is through love through real authentic relationship and connection to assuage the shame and that guilt and the fear and the isolation and I think that profound darkness and isolation is what I hear in you and how fragmenting that has been, how much of a burden it's been. Also the profundity and depth of that and also the potency and power of reconnection and healing that can come recovery that can come by being seen and known and cared for and loved for by another and appreciated and valued and being able to share that connection and I think some of the things XXXXX have raised are new innovative kinds of ways for moral injury to actually be addressed. I have great hope in that and some efforts that are being made right now to address those components of reconnection. Thank you for being able to share the profundity it takes a lot, I can only begin to imagine for someone of your stature to share that with such strength and courage, vulnerability and authenticity at the same time. I know that some members have to go, if you do need to sign off feel free to sign off. I don’t want to cut the general short here.

Moderator 1: We are going to go to the top of the hour, but if I may general we won't keep you too long XXXX maybe just a comment because you were nodding. Just a comment and a brief question maybe.

Student 2: Thank you XXXX and General Dallaire, thank you for your service and your courage for your service and all your humanitarian work that you do around the world. I’m training for a career in pediatric psychology. Just a brief comment on what suzette was mentioning. I do believe that ptsd has multiple components in it and one of the components of course dealing with the trauma and accepting of your past history and working through those difficult memories and associations and triggers. There is also this component of the soul that I want to say which as you have mentioned general Dallaire is only treatable through connection with another soul. With a connection and stability with another soul and that way we can term that connection love. I was also hoping your comment on or speak to the child soldiers you encountered in Rwanda and how the organizations such as the Romeo Dallaire child soldiers initiative works to disarm and rehabilitate soldiers in Africa and around the world. Once again thank you for your time.

Romeo: Thank you. Two things very rapidly and well done. The first is that there’s a lack of spirituality in humanity now. I speak not necessarily being focused on the religion but on a sense of inner spirituality of what the human being is. Because it's not touched upon, it's not nurtured, it's not even spoken of, when you face these extraordinary challenges to every fibre of your being you realize that there’s nothing there behind you to be able to refer to re-establish a balance if you don’t have some sort of spiritual ability to communicate. As you're saying the soul, the being, being able to communicate. And of course communicating with another soul is critical because they exist, we do peer support that is extraordinary and is an underrated tool. We’ve discovered that having someone being able to respond to someone injured psychologically between the formal sessions, when there in crisis and sit there for four hours and drink coffee and not ask one question is an extraordinary tool to help persons work there way through to balancing the hurt the pain to let it out to it's like a pressure valve. Because it is literally pain. It’s a physical and psychological pain and it needs to be let out and it needs to be let out in a safe environment. Peer support, a uncle, a friend, a colleague that’s willing to just keep their mouth shut and listen and let you empty four hours worth of pain, that is an extraordinary tool. Beyond that, when the depth of your problem, as an example, I’ve had soldiers at my child soldier institute, who veterans who have been taking courses in order to train other troops, because we train forces in Africa and south America also, on how to bring new tactics to handling children without simply killing them as they’ve been doing in their doctrine. We’ve had two soldiers who through their sessions of the month long program through the university and so on, who have come up in front of their colleagues and said I have killed a child. They had not mentioned that to their family, to their therapist, to anybody. Imagine telling someone that you actually killed a child. I mean sure it's in the rules of engagement it's in protection, there’s no other option, but how can you do that? It's only when you find a level far beyond just the interfacing of people, a depth that touches the essence of your soul, of being a respective human being. All humans being equal. Only when you find some other human who can receive that can you alleviate that incredible destructive force that eating away at you year after year after year and making you worse. And so I think that you're absolutely right in that the solution is yeah. Love. Love of a, friendship that is a deep love, a spouse or whatever, but discovering that spiritual dimension that can be interpreted and aligned with something that we use the term love. But without that spiritual depth we are vulnerable to self destruction like you can’t believe.

Moderator 1: Thank you Romeo, before we close… you know in the Netherlands we are celebrating may 5^th^, it is now 76 years after liberation of the German occupation. We had a similar situation earlier today when Angela Merkel was addressing a similar group of students in the same way with covid remotely and it was a beautiful session where students could ask Angela questions. But your dad also had a role in the second world war, could you maybe address that and use that as a closing for this session.

Romeo: My dad joined the army in 1929, so he was a professional soldier and then went across in 39 and came home in 1946 and did the European campaign with an engineering company attached to the, part of the Canadian army and fought through holland and ultimately in [inaudible] found my mom and married and I was born as a result. So dad came back and spoke of the war only once to me and that was when he visited me when I was serving in Germany and he had seen the Canadian army convoy go through the German village where I lived. And that night he told me everything that happened to him during the war. And he was horrifically suffering from ptsd, alcohol, lots of alcohol physical and so on it was terrible. He got caught up in one of the worse scenarios, he was part of a Canadian bridge company that was moving forward to support the British to put bailing bridges across on their way to [inaudible] and they were ambushed and they lost a lot of casualties and he mentioned in dispatch, but the destruction on the human mind has ramifications of enormous significance on the families and that’s the side that’s too often forgotten. We have now seen teenaged children commit suicide because they can not live inside a family with a member who is so volatile up and down like this with ptsd that they don’t know from one minute or another if they are going to be slapped or laughed at or whatever. There is no real good system out there to explain what has happened to their dad and or mom and get the proper support because the countries not at war and they don’t understand what has happened.

Moderator 1: Thank you, we are grateful to the Canadians and others who helped us in those final days, 76 years ago.

Romeo: Well, my 27 cousins in Holland are also very thankful. They are good guys they send me cigars every now and again.

Moderator 1: I think we are about to close, before we close maybe if you have an urgent question or something and I know that we haven’t been able to give each of you the floor. If you have something you got to die for you don’t have the opportunity to speak to the general in the near future. Please raise your hand now take the opportunity if you wish because he is still with us for a few minutes, do I see a hand? Two more questions: is that ok General?

Romeo: Yep

Student 6: Sure, thank you General Dallaire for being here, it’s a real honour to be able to talk to you. I just wanted to bring up the study that was done in 2014 out of the university of Geneva and they looked at epigenetic markers of stress responses with mothers who were pregnant during the Rwanda genocide. They compared them to tootsie women who were living in Burundi or who were not affected by the genocide. What they found was that the kids of these mothers had a very blunted stress response and they had a very dysregulated stress response, I was wondering if you could just touch on your experience with some of the survivors and maybe more importantly some of the kids of the survivors who may not have been alive during the genocide but that sort of transgenerational effect that your sort of touching on where the family could be affected directly but there is also this sort of physiological aspect and genetic aspect that comes with it. If you noticed anything or experienced that at all?

Romeo: I have not seen too much although I must tell you that the current young generation the ones born afterwards, and a few years after that, are quite demanding on getting a clear picture of what happened in order for them to be able to bring closure to what they lived in the family. That I think has been long and coming, I mean we are 27 years since, and maybe it’s the sort of social scenarios that have been going on with [inaudible] and the trials and all that sort of stuff. But I found that there was a deep seeded urgency with them now. They are in their 20s, of wanting to bring closure of what they had to live through within their families of people not talking, which was the worst part. People didn’t want to talk about it, what happened to uncles and aunts, refused to talk about, of tripping over the vestiges of the genocide without being explained, without being coached through that and all though there was information provided it still didn’t give them the depth of what it really meant to their society and to ultimately themselves, into the future, and so its only coming out now. Whether that’s been delayed, as you say physiological dimensions I don’t know, at least it's putting the survivor generation on the spot of trying to explain it far more clearly.

Moderator 1: Thank you, There is a lot of intergenerational transmission of trauma knowledge at the U of A. Maybe you're aware of [inaudible] work at [inaudible]. XXXX.

Student 1: Thank you general Dallaire for speaking with us today. Such a enlightening conversation to have with you. I wish I could have this every week with you if it was possible. I was speaking with my dad that I was going to meet you today and he remembers you from his time, from the limited media coverage that he got back in the day in 93,94. And that’s kind of where my question was headed, I had more of a simple question. What would Rwanda look like if it happened today? You spoken about the interconnectedness of our world today, there seems to be a lot more global activism through the internet, but at the same time, its sort of juxtaposed with this idea that today we are inundated with so much information and news, and a lot of bad news, and I feel like from a moral code or ethical aspect, this may sound bad, sometimes personally I feel desensitized to all this bad news. I was wondering if you could sort of speak on how media is covered today and if it would have made a difference in terms of how Rwanda was handled in terms of its diplomacy?

Romeo: Well let me put it this way, I think that the era in which we are in is revolutionary. It's revolutionary by the ability to travel so easily, really. It’s revolutionary by the technologies that have permitted the communications to be able to be real time world wide, I mean we can talk to anyone on earth nearly. And so that angle, is a whole new phenomena to a country in the middle of Africa where there is a civil war that ultimately ends up in a genocide of 20 odd years ago. The isolation of that time permitted the extremists and the international community to act the way they did. One, the extremist to believe they could get away with it, and damn near did, and the international community to get away with not intervening because they couldn’t get all the information, they seemed confused, it wasn’t clear, should we intervene. And remember the Somalia scenario that just happened months before with black hawk down that put the world power in an absolute mode of not wanting to be involved whatsoever. So the difference today is it can't be camouflaged. Even in China where they are trying to camouflage it with the Uyghur, they can’t camouflage it. The question however is who’s going to push the button to get things done. I am of the firm belief that it is not the generations above you, I don’t know how old you are, 25,26 maybe, working on your moustache I see.

Student 1: trying [laughter]!

Romeo: You guys are the ones who hold the key to the future. Because you master the global capability of coalescing the youth of the world into pushing humanity into a different frame in regards to peace, security, equality and in fact ultimately the serenity that humanity needs. You can do it because you’ve got tools, so as the same way you’re saying it makes you numb on what it is, on the contrary it could be used if the maturity of those who are using can grasp the fact that they can have significant influence. You can become activist like we could never be before. I mean real, real activist. Real time, worldwide, you can establish that. You can in fact be the expression of the balance of power in all of the democracies of the world, why? Because the demographics are on your side now. The countries with 15,16 year olds who are 50% of the population. But the demographics are not just the numbers, all they count, it’s the ability of force multiplying those numbers with the technology you have to reinforce each other. Let me give you an example, my country last election 2.9 million votes, that belong to the 18-26,27 were not used, not used. Those votes could if they were used change the face of politics in my country in one election. If the youth decided to coalesce and create their political party to push their own political agenda to coalesce that, they could change the face of politics in a democracy in one election. Why? Because you hold the balance of power. Your votes have never been counted. So those votes are the balance of power, it's yours. All you need to do is in my opinion is recognize that you are without borders. Don’t limit yourself locally. If you need an instrument, create and NGO, it costs nothing. Ultimately I do believe that the NGO community will coalesce one day and will in fact become the significant power of influencing public opinion and policy. Because they are not going to stopped by borders and countries that are still working under this sort of [inaudible] construct are going to see that they are being hit by coalitions that are global, led by you guys. So get off your butt and do something [laughter].

Moderator 1: Thank you so much. XXXX.

Moderator 3: On that point General, I think this sort of summarizes what you just said. We have a tendency to look at these young people and say, you are tomorrow’s leaders. I just want to confirm what you just said, these are not just tomorrow’s leaders, these are leaders today. You don’t have to wait until your 40s to become a leader in the country. You can be leaders right now, I think that’s an important point to raise at a particular in light of what’s going on right now in places like Myanmar I thought I should mention Myanmar because we keep saying never again to these atrocities and we saw what happened in Rwanda and [inaudible] and it continues to happen. The problem we have now is you have a case in Myanmar where the genocide is like a slow genocide, and is not that different from what’s happening in the northern part of China with the Uyghurs, its also a slow genocide going on there too that we need to confront. So I think today's leaders, you guys, are today's leaders now, you have to find innovative ways of addressing these problems, so when we say never again, it becomes more that just simply rhetoric. I think this is something Romeo Dallaire has been trying to do for a long time. To change that narrative and make sure that we never again, suffer the atrocities that we have gone through in the last half century.

Romeo: Well done. I had twenty years of apprenticeship to become in the position of being in a position of influence, you guys don’t, you guys are lucky if you get four or five years before things change so much that you got readjusted. So you must shape the future and not survive it, not try to survive what we are creating, but you got to change it. It is a revolutionary time and it is now, and in so doing you will in fact bring new ideas and new concepts, a new lexicon to how we try to face the future and face the problems that we are facing. And there’s a maturing that has to happen, let me give you an example. A few years ago, I was involved with the program of going after Joseph Coney and a whole bunch of university students coalesced and I was involved with that. And we picked, they said that nobody wants to stop this guy from doing the child soldier recruitment in Uganda and he’s destroying, so were going to stop him, so they got together and collected 70 million bucks, and they were going to pay resources to stop this killer, this terrible, with the large resistance army. Well they got the money but they were not able to implement it because they didn’t follow through they didn’t sustain the effort, and in so doing, Joseph Coney became more popular because the whole thing was done electronically and coalescing so he became even more known by this failure of stopping him than he would of if it hadn’t been initiated in the first place. So there’s a maturing that has to happen with that tool, that you can do, because you can master it. So do it. And that’s it.

Moderator 1: very empowering what you just shared with us, and incentivizing and beautiful words and wisdom there. Thank you so much general. After you, we will let you go, XXXX will address the students, but we will let you go. Thank you again, and I think I’m speaking for all of the students, they’re all nodding, lots of love to your wife too.

Marie: Hello

Moderator 1: this is a beautiful picture.

Marie: Thank you

Moderator 1: This is the embodiment of love, beautiful, wonderful words the General has shared with us, about the ability you bring together, speechless. I’m sorry

Marie: You know he wants to live, he wants to, well eternally, he wants to live eternally with me, so I’m working on it [laughter]

Romeo: 25 years and the military family support center helping military families whose soldiers were coming back from wars and so on and help build the programs to assist them, so she is very very conscious of the impacts that and veterans

Marie: But I’m with the most fantastic man in the world

Romeo: I think so too

Moderator 1: Wonderful thank you so much for being with us.

Romeo: All the best to you.

Moderator 1: Bye

Moderator 2: If we can just linger

Moderator 1: Ok thank you take over for a minute, we won’t keep you too long, we know that time is precious. This is touching really, I didn’t know he would call up his wife and share that. After he just addressed love [inaudible] you know very personal things here. That’s very meaningful that we were able to be present to that. XXXX, you want to address the students before we take off.

Moderator 2: Yeah just a couple things, I want to get your feedback, one I’m going to hear your feedback, and if you wouldn’t mind writing a reflection and sending it to us. We are going to be creating a book for the General of the lectures that will be occurring in the fall. We were hoping you would be willing to write some reflections on what you just experienced with the general. How it touched you? The highlights that you had from  it, that you could send them to us, with your consent we could put them together in the book that we can leave the general as the legacy of the time he spent with us. I want to hear from you.

Moderator 1: yes briefly a few minutes to share some thoughts or observations or whatever you weren’t able to express since…

Student 5: Of course, I think it was such an inspirational session and you know even though my question wasn’t to able to be asked through his answers and his own expression and inspiration that he gave us, I feel like it was able to give me insight as future clinicians we can move forward in incorporating trauma informed care, how we can inform moral injury and he gave lots of good examples such as peer programs, peer mentorship, and even just the ability to take a step back and just listen without asking a single question. We often talk about in our program the strength of silence, so I think even though we weren’t able to ask all our questions, I think he did a beautiful job in addressing everyone.

Moderator 1: Thanks, he seems to think about journalists that he met in Rwanda [inaudible]…

Student 11: Hello, I really appreciate this event was put together because I think it was done incredibly well so I really just appreciate all of you so much this was really, far more heart felt than I was expecting, you know coming from, a master of law program so some of our guest lecturers are a bit more technical and the fact that general Dallaire opened up in such a deep and emotional level was novel and also heartwarming and also as a human rights expert to me that you know his whole theory, that the answer is love, is really at the root of all of our work here today is right? All trying to make the world a better place. Really he just named the essence of it which I think we dance around far to frequently. So the fact that such a prominent person whose had such an esteemed career and has been through so many struggles and has really seen so much, for him to be able to articulate that was really really powerful. Its definitely going to sit with me for a really long time. Its given me a lot to think about, I’m really grateful for this space.

Moderator 1: Thanks for sharing that, XXXX.

Student 8: It's definitely given me a lot to think about as well, yes its one thing to read about things and to watch documentaries but to actually hear general Dallaire speak is really impactful. Especially the part where he’s talking about the importance of having spiritual depth and not having that leaves you vulnerable to self destruction. Just as a person but also as a student working, trying to work in trauma informed care, I think that was really important for me to hear.

Moderator 2: Thank you….

Student 13: I think it was a very inspiring event, and well although not my questions were asked, actually all of them were answered through the other answers it was really helpful as well. Although I do not have a background in psychology or psychiatry, I really was inspired by the fact how general opened up and talked about how he dealt with disease, and it was really inspiring for me as well. Thank you for organizing it.

Moderator 2: Thank you, XXXX.

Student 9: More echoing what everyone else has said it was very inspirational, great to attend very informative. I was struck by the passion of it and I think it really kind of helped me understand more I guess the post traumatic sense and the moral injuries and the soul aspect of it vs the trauma aspect I think has really affected observation of the topic. The call to action at the end was very inspirational and brings everything into perspective into the role that we have and the things we can do to change and realize that call. I guess the call to action to not allow these things to happen again. It was really great, thank you for putting it together.

Moderator 2: Thank you, XXXX.

Student ?: I would just echo what the others have said so far and say a big thank you to the organizers of this event. It was a very inspirational talk and being in a masters of law it was really nice to hear a heartfelt talk and very inspirational, and yeah just really appreciate general Dallaire opening up and telling his story and taking the time do so. Thank you to the organizers and everyone who asked the questions it was really interesting.

Student 3: It was incredible. It was a really great hour of just learning lots from general Dallaire. A couple of the points that really stood out to me, the point about how national self interest and what that looks like in our day and age right now, especially now coming from a healthcare perspective the pandemic and how I guess some of the nations navigate through that and the crisis that is currently happening. Also just with him speaking about his institution and that kind of thing, about challenging myself and what kind of legacy I want to leave behind, we as a nation leave behind, we as a generation what kind of legacy and how do we want to move forward. The last thing, just kind of a challenge because I’m sure everyone is feeling very inspired, very passionate, that call to action I guess, how do we balance that zealousness and almost even a little bit of naiveness with tactfulness and maturity he was talking about.

Moderator 2: beautiful questions, XXXX.

Student 12: Yes, for me as a physician it is very easy to prescribe pills for everything also for ailments of spirits. But and by that it might be easy to overestimate their importance but it might be that connection to oneself and to others might just be the final solution in ptsd for example.

Moderator 2: a profound statement and an important one.

Moderator 1: thank you, XXXX.

Student 6: I really thought just that picture at the end was very telling. You know its this beautiful couple and if you didn’t know the context of his background, you wouldn’t think this is someone who has experienced genocide, and it tells you two things. That you don’t really know what someone has been through, but also a perfect example that you can recover and still live a normal life even if you go through these horrible things and it gives us a lot of hope. And one of things that we are trying to do here is to find a solution to the horrors of life that people have to experience and hopefully don’t have to suffer through that their entire lives. I think it was a very nice example of that success story.

Moderator 2: Beautiful, thank you, XXXX.

Student 4: I found it really wonderful to see that some sort [inaudible] to find peace, because I read his book where he explained all his suicide things, and how he struggles with everything, I found it very beautiful in the end with his wife that he found his peace and that true love and true connection, yeah he was able to move on and just wants to live now. Its really inspiring for me.

Student 10: Yes I think it was just quite remarkable to hear a military man of his level of distinction speak with such tenderness but also such a struggle. You can see he is still struggling with this and I think that was so important to humanize these things. What really stood out to me as well was that, and I’m amazed to hear him talk about, this importance of this spiritual awakening to our global interconnectedness and cultivating a real and genuine love of humanity. And I think that is our true legacy to future generations. It is not the wealth that we create in our country, it is that. It is that love of humanity and love of life. That should be the focus, so that was amazing to me thank you.

Moderator 2: Thank you

Student 10: we should have had him for much longer. I thought it was going to be three hours.

Moderator 1: sure

Moderator 2: there is more to come.

Student 2: Thank you very much to the organizers again for this incredible opportunity. I have to, I think I was watching Dr. Vermetten’s face there at the end when he introduced his wife, and I think we both shared an emotion simultaneously where I almost teared up I couldn’t I don’t know how to explain other than blissful the image that I was seeing on the screen. The part that I would like to reflect on the most was the fact that he kept on bringing up this idea that connectedness is ultra important in dealing with ailments such as ptsd and I don’t want to limit it to just ptsd, but we can extrapolate the importance of connectedness and love and relationships and support to all mental health. At least in my field of pediatric psychology I would like to bring that down from a macro level of global connectedness to a micro level and how we can continue to reiterate in psychology the importance of this systems perspective. We shouldn’t be treating the individual, but we should be treating the family, the school the community, the globe. So I would like to reflect on that and how I am very proud of us as humans moving away from that purely medical hierarchy of treating the individual to a more systems, spiritual, connectedness perspective, how ever you want to put, at the end of the day we are one large organism living on this rock.

Moderator 1: Nicely put…

Moderator 2: I just want on a scale of zero to ten how was this experience for you, 0 being really crappy, 10 being really great. Just show of hands…

Moderator 3: I know that general Dallaire probably wouldn’t remember this but on the 3^rd^ of December 1997 I sat with him on a bench in Ottawa because I was invited by who was the foreign minister of Canada at the time to witness this signing of the land mines treaty. This is a very important treaty that Canada took the initiative, it's called the Ottawa treaty for that reason, candida to ok the initiative to implement because land mines are more dangerous to people after wars are finished than during wars. One of the things that I felt sitting next to him at the time was that he was still in the depths of despair. It was still his sort of darkest moment. He was still considering things like suicide. I see this transition of this man from 1997  to now has been a major transformative event in his own personal life because I think he has moved on beyond that dark moment and he has a lot more hope now than he did then. Even though he didn’t remember this, I saw In one of his books a reflection about him being the confronted with a ak47 and he had the choice of blowing that individual away and then realizing that the person was a child. He had to make a decision a split decision about taking the life of a child with an ak47, and I think one of the things we talked about because I was working on my child soldier project at the time, was the importance of never allowing this to happen again. Never allowing individuals to recruit children into war, because it poses a moral hazard for individuals like him, soldiers that have to deal with this sort of thing on a personal level. I think that was a very important point in my own development too as a scholar a person who works in this field of child soldiers. I think the admission that he gave today that you young people have to start acting now rather than forty years from now or 20 years from now, you need to act now in order to bring an end to these atrocities that are happening around the globe. They are still happening, they are happening Myanmar, in northern China, different parts of the world. You need to be able to act now rather than when you're much older because time waits for no one as the saying goes. I think that’s a very important admiration he gave us today. Thank you very much students for attending and your input. Thanks to XXXX for putting together very succinctly the discussion we had before this event because I can tell from his responses that he had actually taken the time to go through those questions. So even though we might not have had the time to ask yourself the questions on them today, I’m sure he was able to reflected on those questions and was able to form answers that address most of them, so thank you very much.

Moderator 2: thank you, XXXX

Moderator 4: thank you. I’m a clinical psychologist I work mostly with kids and families and a prof at the u of a. I think you what, I think I was before, coming down to connection so self and to others is important, so important. I know in my field as … mentioned in his own way, some times we stay right here, and if that’s where we are staying then we are missing the boat. My soul was touched today and I was not expecting that. So my personal challenge and all put it out there to all of you as well. My goal today is to touch another a soul like I was touched this morning. That’s my personal challenge and I lay that challenge out to all of you and I think if we can intentionally do that, daily, I just wonder. Thanks.

Moderator 2: thank you XXXX. XXXX.

Moderator 1: Well yeah, what more to add, what I love is this here, that we were able to in covid, to organize a thing like this and to incentivize young students. I love doing that and I love seeing how your brains are wired up and the wisdom that you put in being here reserving time to create something together that is better than the individual parts. That is beautiful, sort of wonderful and if I think of you know every time, I’m a colonel in the Dutch armed forces, working with veterans so I see a lot of veterans every day. And when I teach I show this clip of Romeo Dallaire where he says “ what I can not stand is the loudness of silence” so I know that clip, and I’ve never had the opportunity to ask him how that was now for you thirty years later? I never had the opportunity to I couldn’t even dream, I didn’t do it last time because it wasn’t there to ask him, and this time I felt prepped up so here’s my chance, and that came the answer about his wife and about love and that carries him through the night. And he was vulnerable enough to say, as a psychologist I’m very intrigued on how people do that and he says, I’m still struggling with that, and I still live through the atrocities but with my wife being there and the love that I had there that’s my ability to cope and that touches me profoundly and I can think how I’m going to add that to my lecture when I lecture about ptsd now I can add, I spoke to the general 30 years later, you know the question that he gave. Its going to be on lectures, I’m going to make a slide, I think I’m going to make a slide of him and his wife. Thank you so much for being here and also XXXX for moderating the last peace, because the piece that he was with us but this reflection part is as important because it gibes us the opportunity to reflect on the session and this is always a very good moment that it sinks in. so suzette, you close the session please, as the last, I like how you did this, everybody you gave the word. Suzette is beautiful in a way she organizes these events I must say on behalf of us and it was beautiful that you were with us. Too bad XXXX had to leave a little bit earlier. XXXX to you to close, because you also have a assignment to the students briefly you referred to that already earlier.

Moderator 2: so an invitation there’s no expectation, nothing that we ask of you this is an invitation for you to be a delegate and to enjoy this moment. We will have some lectures in the fall so in September through November we will have a lecture series of about eight lectures, we will put you on the list so you know when they are. If you feel up to it to even right a word or short two lines or paragraph or whatever you like in response to this. We will be putting together a book for the general and we will selectively go through it. We will also give him this recording, nothing else will be done with this recording unless we ask you for it. We might take a quote or something in here for him from one of you as we are anticipating the fall lectures, something like this was really great, it was great to hear the general be so heartfelt… the importance of humanizing or the importance of connection that we might grab as we promote. Otherwise my word to you is think deeply and let it sink in, be touched where you need to be touched, be inspired where you can be inspired, be invited into action and where you feel called to respond. Otherwise thank you for being with us, know that your questions were answered, your questions, everything in the chat was sent off to him. Rather than taking the time for each of us to ask the question, he received them in advance from what we prepared Monday. Thank you each, Enjoy the day and all the best for the rest of your term and rest of your summer and we  will hope to see you in the fall, thank you XXXX for everything you do to make this, thank you all.

Moderator 1: alright, good luck all and thank you for being here.

**September 15, 2021 Session 1:37 Duration**

Moderator 1: so we’re a group of…yup now you can say got it. Now we’re a group of 18 now and uh it is five after five local time where I am in uh Leiden this time not in Utecht, but in Leiden in Netherlands and um I welcome the General uh dear General. We’re welcome to have you with us for this uh for this session um and as we uh scripted uh briefly before the session, what we’d like to do is uh for the students first to um call out who they are just your names and where you are and anything particular not eating too much time away of the discussion that we’d like to have but at least we can hear you. I’ll call your name or  XXXX will call your name and then uh we’d like to circle then to the General and the General will lay out some introductory remarks uh as he has been doing for the previous sessions and then we’ll in engage in the the questions that uh we have collected and forwarded to the General after this, after our meeting yesterday at yesterday at uh evening uh yesterday morning whenever it was wherever your time zone was. XXXX am I, anything that you’d like to add or maybe you could uh uh let your voice be heard as well? And your again muted but that’s oaky.

Moderator 2:  just thank you everyone again for taking the time out to uh come join us and General Dallaire it’s an absolute privilege having you with us. Um maybe we can just start very quickly for the sake of time just to hear everyone.

Moderator 1: yeah. Okay XXX if I may ask you first?

Student 1: hi everyone my name is XXX. So exited to be here. I am located in Edmonton, Alberta Canada.

Moderator 1: thank you XXX. And uh XXX?

Student 2: well hello everyone. My name is XXX from XXX and I am located in Utretch Netherlands.

Moderator 1: than you so much XXX. XXX?

Student 3: Hi I am XXX. I am located in Edmonton, Alberta and I just wanted to say thank General Dallaire for coming to talk to us.

Moderator 1: wonderful and XXX. Hey XXX you were not with us yesterday but please

Student 4: I was not yes. Thank you so much so my name is XXX and I am currently located in Calgary Alberta and thank you so much General Dallaire. It is a privilege to be part of the conversation.

Moderator 1: thank you XXX. What are you studying XXX?

Student 4: I am currently studying uh education.

Moderator 1: thank you. XXX? Hi. Always good to see you.

Student 5: good morning. Good morning General Dallaire. Thank you so much for joining us. And my name is XXx and I am from Edmonton Alberta Canada.

Moderator 1: thank you XXX. XXX?

Student 6: hello my name is XXX XXX and I am calling from Edmonton Alberta as well.

Moderator 1: lovely and then we have XXX.

Student 7: Hello thank you general for being here today with us. Um I am calling from London Ontario.

Moderato 1: great thank you XX. XXX XXX?

Student 8: Hi XXX XXX. XXX Science at U Alberta. Thanks very much for for doing this and inviting me.

Moderator 1: good to have you. Uh XXX?

Student 9: hi I am XXX. Currently in Edmonton Alberta. Thank you.

Moderator 1: lovely and then we go to XXX. XXX please?

Student 10: Hi I am XX. I am also from Edmonton Alberta.

Moderator 1: absolutely and then XXX.

Student 11: hello I am form XXX and I am from Calgary Alberta. Great to be here. Thank you.

Moderator 1: lovely and then a very familiar face to you I think uh dear General. XXX?

Student 12: hello General

Moderator 1: you need to tell where you’re located XXX.

Student 12: oh he knows but XXX Nova Scotia.

Moderator 1: yeah there we go. There you go. Not driving today. Okay then um XXX?

Student 13: Hi my name’s XXX. Opposite from my name, had to sign in from a different computer but

um I am coming in from |Edmonton Alberta.

Moderator 1: thank you XXX and we have XXX?

Student 14: hi my name is XXX. I am calling in from Leiden, the Netherlands. And thank you for this opportunity.

Moderator 1: thank you and then we have XXX

Student 15: I am XXX and I am from the Netherlands and I am a PhD student um uh working on veterans with PTSD.

Moderator 1: Thank you. And XXX is not with us yet. XXX. Is there anything? Okay, for XXX?

We had a XXX and a XXX. Okay, thank you for, for calling your names and where you are. I am

happy now to return it or to invite the general to open our our student engagement session unless I

forget something I see your smile XXX. Is that meaning anything that I forget something?

Moderator 2: No, we’re good

Moderator 1: No. Okay All right. Dear general may I give you the word. And by all means, no

introduction. Absolutely. But you’d like to make some opening comments, to distill the beauty. As you

heard, these are students from a variety of places in the in the world, Canada and the Netherlands,

studying heterogeneity of of things, ranging from what we haven’t  said that we have seen it in the mail

that we sent to you yesterday.

06:05

RD: Thank you very much, XXX. And XXX, and thank you also, to all you students, I love the term that

have taken me are taking the time to spend this hour together. And I am going to give just a couple of

comments to cover some of the possible grounds that I hope are of interest to you, but mostly I am

seeking questions from you, because this is an interactive exercise in which your concerns with you are

trying to, to understand or know more of, is what I hope to be able to respond to. And that’s the that’s

the the ultimate aim of Are any of these such exercises. The the first arena that I wish to bring, again to

the attention of all of you is the arena in which we are now facing significant crisis is and that is the

catastrophic failure of stabilization and security, missions and and ambitions, the world in countries that

are in conflict and trying to pull themselves out of conflict. And certainly Afghanistan is is a perfect

example of a debacle. For a variety of reasons, which certainly stem from the fundamental and not

understanding of what the people of Afghanistan want, and ultimately, what their ambitions are all the

way through to in in Asia XXX is I would say, of big powers with with peripheral powers like Pakistan, in any peace process, that could have any chance of lasting 20 years is an eternity for a politician. In our

era, they I mean, they can barely survive four years as we’re going through an election here, after barely

two. But imagine that when you are trying to engage in a significant shift of a nation into a new way of,

of establishing the responsibility of governance and ultimately, of respecting fundamental elements like

human rights, the rights of individuals, the cultural respect of the different elements of our society, that

20 years isn’t long enough. And so, why we have this sort of neocolonialism, that essentially says that

we can, we can change and make, in fact, near a revolutionary change in nations, in periods of four, ten, 15 years, even is is the right off the bat setting us up for some of the most catastrophic scenarios,

scenarios which we see Afghanistan turning into, you want to do such fundamental transformative

changes in a society to give it a opportunity to maximize this population, and to establish an

atmosphere of security and the respect of human rights and human beings. treating them all as equal,

and then you’re in for at least 40 years and beyond. And that doesn’t mean that you’re using force

throw that of course not it means that you are using a multitude of disciplines that are integrating into a

a instrument of assisting these nations shifting into a different era,

10:08

we have spent massive amounts on the military side, on the security side. But we’ve done so little on all

the other dimensions of stabilization, from from governance, to human rights to, in fact, nation building

and establishing a capability of a nation to advance and take care of its people and protected in the

human rights dimension, at least, let alone advancing it into an ability to achieve new goals. So the first

arena is one of our all humans human and involving ourselves, in nations in conflict, conflict resolution,

and maybe even trying to prevent conflict is a highly ambiguous, highly complex, and requiring a whole

new set of instruments including a change in even the use of military force, as purely a kinetic

instrument of, of security. But in fact, as a progressive value added element into the stabilization of a

nation. So that’s, that’s a whole reeducation exercise to be done, and several disciplines and an

acceptance, that you’ve got to integrate these different disciplines into one new instrument of assisting

nations to advance. I think the other arena that is of significance to me is, is the arena of the impact of

many of these failures, and those who are engaged and see that their efforts and the price of sacrifices

that they’ve committed to these missions, these tasks have been essentially for not or nearly for, not,

not necessarily because they haven’t, individually or collectively even performed, but in fact that the

entity in itself was not, didn’t have the depth to be able to implement, as XXX will easily say to all of you,

we can spend years training people. But if there’s no depth to the ethos of what that military is, there is

no instrument to which it can commit itself to the government and the people in a way that the people

see it as a value added to their security, then all you’re doing is training individuals that ultimately will

they want to fight will they want to commit and sacrifice themselves. And so many of those veterans,

both a military, both political and even NGOs, are going through some very traumatic times right now.

One trying to save people that were that they work with, and they know, are at risk. Speak up against

that, of course, now, but it’s happened in other areas they haven’t done to me when I was in a Rwanda,

as well as trying to come to grips with, in fact, this, this, this feel of not of guilt, but in fact, of horrible

wastage of loss of sensing of abandonment of a task and a commitment that they did, and which

included some of them losing their lives, of course, and many of them have seen their families

mortgaged and themselves mortgage for life by the

13:52

injuries that they’ve sustained physical, but one injury that is so much more complex and the mandate,

and that is from PTSD to moral injury, the depth of the moral dilemmas that we live through and to see

that ultimately, as we try in the field, to assist in meeting some of these complex and ambiguous

mandates, that ultimately, we don’t necessarily succeed, or we thought we succeeded only to find that

other elements that were to participate were never there. And so those are embracing themselves into

an overall reassessment of the era in which we find ourselves. We’ve stumbled into it. And we still seem

to be ad-hocing a lot. We still seem to be crisis managing, and ultimately, we still seem to be searching

for what is the instrument of peace? What are the principles of lasting peace, which I am now engaged in

with a commission Under the interpeace, at the Geneva, as we are trying to look at what is what are the

principles of lasting peace, because so many of the conflicts we’ve seen, and peace agreements have

crashed by very bad implementation short term, and ultimately no buy in by the populations themselves

the result. My last comment due to, as I am throwing these elements out to you for, for questioning and

for pursuing is the the, the grasping of what cultural, cultural change is, in fact, cultural reform, cultural

realignment, cultural acceptance, that there are elements that simply are changing and others that are

a basis upon which to change. And so it is not by subjugating the culture of an organization, it’s the

ethos or a society of ethnicity or a group that, in fact, by subordinating them, you think that you’re

actually changing things. On the contrary, I would say that you are emasculating the members of that

cultural group, that organization that thinking, in masculinity, from being able to participate in whatever

change reform, adjustment is needed, for them to continue to progress. And to advance and to permit,

the generations that are coming, including the one that’s there now, to be able to maximize their

potential. And so those of you who are students, and probably those of you under 30, anyways, if not

25, I consider you to be part of the generation without borders right now. And with the revolution in

communications, and social media, and so on, you have instruments now, that empower you to bring

along significant change and engagement. And, in fact, permit us the elders to hold you accountable for

engaging in shifting the world, and the whole of humanity, which soon will be able to Skype, in fact, the

whole humanity into a new era of respect of individuals human rights, and that the frictions of our

differences do not lead anymore, to conflicts where we try to destroy each other, or our own cultural

frameworks. So that’s a bit of a feel for the exercise, and I hope it will instill in you an interest. Thank

you.

17:54

Moderator 1: That is very important. Three, three very important arenas that you outlined. And they all they all have a call to action in themselves. I think I don’t want to comment myself, but I feel that I would like to give the word to the to the students really, and and just in any order, but maybe I’ll start with you. If you wouldn’t mind, XXX, if you wouldn’t like to bring bring your question about to the general to Romeo. And then we’ll we’ll engage and we’ll start and we’;ll see how how the questions could lead and be tied into another end, please feel free to jump in, open the mic or you can raise raise your hand or make any signal or so if you want to do to add so that we don’t speak in each other’s mics. But yeah, that’s the

sort of etiquette that we could use. XXX, if you want to start first, please go ahead.

18:51

Student 1: Sure, and you’re speaking about the question we kind of started talking about yesterday. Is that correct?

Moderator 1: Absolutely. Yeah.

18:56

Student 1: So the question that I was kind of thinking about over the last week, and it’s been on my mind for maybe the last year and a half already with current current political situations, advocacy movements, ismy concern for first responders and military veterans. And my specific question, I guess, was that you

know, just like the Vietnam vets coming home, just like you coming home, there’s often this shame,

blame, social betrayal that comes upon these people, you know, saying how could you have done that?

You’re doing it wrong, you should have done this, this this. And often that can kind of layer on top of

current PTSD that they already have from the service that they’ve committed their lives to. So I was

kind of curious about, you know, what is the impact on PTSD, moral injury, when we have media,

everybody is so connected now. Layering, layering, that social betrayal on that and how can we

respond? onto her address, you know, this type of social betrayal to try and mitigate that or prevent that

from happening or somehow protect our first responders, I hope that makes sense in formulating that,

in my mind is

20:15

RD: I think you’ve, you’ve touched a very interesting dimension of of, of people who have committed

themselves to service of others. My father, when I joined the military, he told me remember, don’t

expect anybody to say thank you, because you’re there to serve. And so already, when you

volunteered, to, to these engagement that you find yourself committed, far beyond simply a job or a sort

of task. Often you find yourself in nearly a vocation of your life, and to find that to be assaulted by the

by the same society that has sent you into doing these tasks, and exacerbates significantly and

deepens the wound. And I use the term wound, these are injuries, the psychological impacts that come

back, that you already feel. Because there’s no more or the era of winning the war and having big

bands and parades, and so on, and medals and everybody is now moves into reconciliation and

rebuilding, what you have is these conflicts that you’re never sure whether or not it’s been solved. If it’s

in, in a positive seller’s solution mode, or whether or not it’s regressive. And you can find yourself

caught by surprise, just like Afghanistan seems to have been, although the world of science, I was a

young officer during the Vietnam era, and I remember how the that war had a catastrophic impact on

the American society. I also spent a year with the US Marines, and all my classmates were all

graduates at the same time. And Staff College there who had done at least one if not two, one year

tours, and all were physically injured, let alone psychologically impacted by that. But I also live long

enough to see that the same veterans that came back from Vietnam, and we see from other missions,

be it the Yugoslavian mission, maybe even by my when I was in Cambodia, with the Cambodian

mission, and with the Rwanda, that even though we come back with those injuries, and we face adverse

impacts from our society, that can change. And it is that change that gives some comfort, to addressing the sense of guilt, the sense of having abandoned the sense of not having finished the job, and and ultimately having been even betrayed, by those who essentially, by having a society See, clear, much clearer than what I am afraid the media and often, many opportunistic writers have described as what happened. I’ve seen how Vietnam veterans in the United States, in held in highest regard for what they went through, under the the circumstances of the political scenarios in which they stick they served and gave so much. I

have myself seen coming out of Rwanda and facing the international tribunals, and having to not only

try to get people to be put in jail, but also defending the actions that are taken. I’ve seen us accused the

UN of having failed when we know that, in fact, it’s all the individual states that made up the UN that

failed to give the UN and ultimately me in the field and my end troops, I had the ability to prevent that,

that genocide, let alone stop it when it was going on. Because it was not in the self interest of the

nation. So the depth of these wounds are greatly exacerbated by In fact, how the society sees you

upon upon your task. Because one of the terrible elements of living through these very complex and

ambiguous mandates and also seeing some of the most horrific actions that humanity can do which

attack your moral fiber from right down into its root. One of the most terrible dimensions of that is, is

that we don’t seem to be able to explain clearly what, what happened. Because whenever we might try

to explain it, people just can’t handle it. People just don’t have either the time, the patience, nor the

ability to see the depth of the injury and and what we’ve seen, and the impact of what happened in the

field. So I think your your question is, is most most pertinent particularly now, I mean, we sent nearly

40,000 troops through Afghanistan. And I am sure, to the men and women that have made it through

Who’ve suffered, the the injuries that they lived through there, and the sacrifices of them and the

families, that they are hurting. They’re hurting again, and they’re reliving it. And I can tell you, as my

wife and I have been involved in trying to save Afghanis right now, is that what this thing did, was just

brought me back 27 years, when the Rwandan genocide started, and rushed to the airport, when all the

white, you know, Europeans mostly, were running to the aircraft with with the golden ivory and dog, and

leaving behind the Africans that even in some cases, raised their children to be slaughtered. You got a

real problem of trying to face the reality of our times. So that’s my short answer.

26:50

Student 1: Thank you, I really appreciate that. I feel like it’s um, it’s hard for us who haven’t had our our boots on the ground. Like I have some skin in the game because my partner was a reservist, and he’s police now. So I have that bias of, you know, there’ an extra target on his back. I see that. And that concern for other service members, but I definitely see my veteran clients being very impacted by the current situation in Afghanistan. I appreciate your, your comments.

27:17

RD: Thank you. And I liked your comment about boots on the ground. I think that the I think the generation without borders should have as a, as a sort of a rite of passage out of college or technical colleges or even High School, that they should have a pair of sneakers underneath their bed, soiled on the earth of a country developing country where they can see taste, touch, feel, smell, what is happening to 80% of humanity, and bring that back, and that that keep the flame alive in them to make change into the

future.

27:52

Moderator 1: So will thank you, we’ll hear a flurry of other topics and questions. There’s never an array. We’re already at the bottom of the hour. Please, if I may call now for XXX. XXX. Is that. Okay, if you wanted to bring forward your question?

28:07

Student 14: Yeah, of course. Well, as we discussed yesterday, my question was actually very similar to XXX. So It’s been mostly answered already. But there’s something else that I would like. If that’s okay, go ahead.

Moderator 1: Yes, go ahead.

Student 14: Yeah, so I was, I was thinking about the, the limited mandate that you had, and during your mission in Rwanda. And this seems to be there’s a, there’s a parallel with the Dutch mission in XXX. Where there was also a very limited mandate, and not enough soldiers. And this resulted in a genocide. So there’s a lot of parallel between these missions, I think. And I was wondering, for you, how does this mandate influenced how you were able to cope with what happened? Because I can see it going. many ways, I can see feeding into a feeling of hopelessness about the situation, but I could also see it maybe as strengthening one’s feelings about one’s own actions, because you really couldn’t have done more than you did. And

29:38

RD; I think your question is very first, because because when, as an example, as a field commander, when

you are in command, you are responsible for your mission, success, and of course, all those who serve

with you, and so you, you simply can’t get away. With saying I did the best I could, that simply doesn’t

wash with you and show you live with a constant sense of guilt of not having accomplished the mission

and the responsibilities which are of responsibilities of life or death. In in the field that can can drag you

down, there’s absolutely no doubt. And until you can balance that out with an optimism and an

engagement into the future, it can essentially lead you to what this injury does, at times, and too often

do is to a fatal end due to suicide. But you know, the nation’s yours mine, middle powers, even big

powers, they commit their self interests discussion into whether or not they should be involved in

protecting people. And it is very difficult for the political elites of our nations who are risk adverse, and in

fact, very, very accountable to a population that is often ill advised through some not too in depth media

information, or information from the government itself. To understand that they’re going in with maybe

less than optimum capabilities to solve the problem that they are sort of accepting, often reluctant

reluctantly, to, to go in. The fear of casualties is just one overriding one and certainly after the Somalia,

debacle with the Americans in 93. When those Rangers were killed, and Bill Clinton change completely

the the outlook into peace and engagement in protecting civilians in conflict zones, to what not wanting

to be engaged at all, what is the XXX is Director 25 that he published, we we can see that nations

are dabbling at the world and not committing to the world. They’re not committing to humanity, yet,

they’re committing humans to try to conduct actions that are not only complex and ambiguous, but

nearly superhuman, unless you have the depth that is required. And and the political politicians and the

political elites just don’t have that statesmanship depth, to commit themselves and their nation to

actually not only resolve conflicts in a deliberate, long lasting way, but prevent them. Imagine if we got

into preventing conflicts. I mean, we’re not even close to coming to that yet. So you’re, I am, I am, I guess I am reinforcing your concerns because they are true. And they are of enormous impact. And those who ultimately are in the field, just like your partners, and stand there and find out that you just don’t have the assets, the abilities to make the difference and to implement it. I left command of a brigade group of 5200 troops. And that’s exactly what I needed to ultimately have been able to prevent that genocide.

But I ended up with only 450, who were not equipped and certainly not as trained to do it. So when you

know that, that eats away at you, and sometimes you wonder whether or not you’ve just been set up to

failure and that you’re maybe going to be the scapegoat for others, as the UN is too often being used.

34:17

Moderator 1: I see I see people nodding. Before reaching out and calling for Shannon, just to just a personal, personal question. The students may not have been able to view the documentary, the room, you’re shaking hands with the devil, and if they have the opportunity and what stands out and that’s why I

wanted to just phrase it again, the beautiful quote that you had in that documentary, which is on a

personal note, when a gentleman returns and says what I cannot stand when I can extend is the

loudness of silence. The loudness as soon as I asked you previous previous shooting engaged as well.

Do you want to before we end up with another question, just a reflection on what it is for your wedding?

was for you. And what it is now for you, this is something you can respond to,

35:04

RD: I think… It there is so much of an impact on your ability to come to grips with the catastrophic failure of humanity. And in fact, as I use the subtitle of my book, shake hands with Devil

is the failure of humanity and what was not just the rest of the world not coming to prevent it, let alone

Stop it. But the failure of Rwandans in their humanity to each other, and, and so that, that, that failure,

and the the witnessing there of just doesn’t give you the ability to absorb this within the context of our

cultural and, and sort of ethical values that we, that we hold. And so you, you, you, you fear, you fear

the silence, you fear the darkness, because it screams at you, what screaming at you, in my case was

800,000 souls, nearly 4 million people internally displaced and refugee of that 500,000 were estimated

500,000 were orphans, from the slaughter. And the smell, they the whole atmosphere is is is still alive,

because with time it doesn’t disappear. You still live and can live digitally clear and in slow motion, what

you’ve seen and heard and felt and smell. And so that silence is an opportunity for all that to invade and

overwhelm, and ultimately, can be your self destruction. And so I’ve got over 20 years of therapy, I still

take nine pills a day. And I have found one solution that has been overriding all the others in trying to

solve this, and I get great therapists, you know, great psychiatry, psychologists deal with the with theirs.

And I am not negating at all the assistance that they have provided a professional help peer support to

this. But I discovered only about four years ago, four or five years ago, is love. Love is the ultimate,

ultimate writer upon which our lives exist, not ambition, not ability to succeed and so on is his love to be

and to be able to love another human being that at the end of the day, when your head hits the pillow is

the greatest asset to being able to survive and overcome and in fact, find opportunities and optimism

into the future. And that has been my saving grace. Because if I hadn’t found that I would not be talking

to you today.

Moderator 1: Thank you for sharing that with us Rami, this very, very, very touching.

RD: I my lovely wife is is listening to be also working from home. So I am in good graces here.

Moderator 1: Absolutely. Excellent. You shared that that thought with us last time. And I think it inspires coming back

from your end, very much this year with a yearly. So thank you again, I just wanted to now turn to who

already announced that she knows that I am calling her name XXX. XXX, would you would you

raise the raise your hand and ask us the general your the topic of your of your concern or your

question? Well,

39:13

Student 5: I think you’ve segued into it quite beautifully. As you were discussing the futility and the wastage and

the loss that you’ve witnessed throughout many of your engagements and how you felt that there was

an attack on your own personal moral fiber but that you were balancing it with optimism and and now

you were just describing with love. How have you forgive yourself for things that you cannot control?

RD: You have You put a question to me so profound that I haven’t. I haven’t had such a significant question. Since a young grade five students asked me whether whether I’ve killed How does the soldier respond to that, you’re, you’re asking whether we can self forgiving to for what we’ve done and knowing that lives depended on decisions you were taking on choosing who would live in who would ultimately die as you, on the end of the phone, say you couldn’t send troops and you could hear them being slaughtered. there and screaming. I am not sure that forgiving myself is, is ultimately totally possible. I think that I am eating away at it, and I am handling it. But you know, death is his terminal. There’s no coming back from it. And when you’ve been involved in decisions of life, and death, that, that responsibility, that decision is eternal. It stays with you for your whole life. And so it is a most profound element of your ability to, to stay and wanting to stay alive is to how you can master that guilt, that ability to objectively as best you can see the decisions of the past, but you will never ever erase the impact of those decisions on you. I could be terrible and saying you will never you’ll never erase the blood off your hands. It’ll, it’ll be hidden in the pores of your hands for life. And I think that’s a responsibility and a sense that you must maintain when you had to take decisions of that nature. I think it’s all the more so when people decide on the future of other individuals and other human beings, even though it may be not life and death, but it could be death of their culture, by the way of life. I mean, there’s things like genocide, yes, life and death, but there’s also cultural genocide, you know, that that is critical, and has led to people wasting their lives and dying because everything that they use as a reference has been emasculated and destroyed. And how many women have been abused, and on one side, the ethnic different ethnic groups and ethnicities and communities? Well, Aboriginal community I, I sat on the Aboriginal community, First Nations community in in the Senate. And I still remember the Aboriginal these while they were first nations and Mete. Women came to the Senate committee arguing that they should be allowed to have rights like the men have, and that the we should return to the premises of what was the rights of women in the past, in many of their communities, which were matriarchal. And that, that the infrastructure in the everything they own. It was the women who managed that men went often fought

and got fooled and stuff like that, but they ran the show. So they caught us completely by surprise, but

not wanting to be equal, but wanting to go back to a role that was that was in, in their instinct in their

culture. And that was extraordinary. And the answers that were coming from too many colleagues were

so so adverse to it, that at times I felt like a white XXX, you know, and and in the horror, of

realizing that laws like the Indian act that says in the first line, it’s 100 and some odd years old a first

line says this law has been established to assimilate These people. I am a French Canadian, and I am

fearful of being assimilated into the English media, and probably have been to, to a large extent. So

imagine what you’re going through. And when imagine what women are still going through. And it’s

male dominated world is failing humanity. And it’s high time that we give that a whole reassessment of

women in the, in the social construct of our of our world, from religions, to, to governance, to, to ethos

to our references, ethical and moral references. That’s my short answer on that one. That’s for sure.

49:10

RD: I think that you you touch on a number of points that we finish with Omar Gaddafi, I have been involved with the concept of responsibility to protect that says that the individual human being is sovereign, not the state. It's the state is not an absolute sovereign anymore. It's the individual that's the absolute sovereign. And in so doing, we have a responsibility to protect individual when their human rights are being abused by either the government or by the government not being able to stop that. And so responsibility to protect means that we go and we assist in protecting the civilians. In the case of Gaddafi, there was never the ambition of responsibility to protect To do regime change it was simply protect the civilians and permit an atmosphere that will find a solution between the governance and whatever's creating the problem, and the innocent civilians that are being abused and so on. But the inability of in fact, the nation states of controlling the assets that they commit to that is how we ended up with the mess that we ended up with, that NATO had no control on it, the United Nations Security Council was divided and how it wanted to solve it. And so the the conceptual framework of it was, was misled, the guy who commanded the forces, that which were mostly Air Forces, was a Canadian, a colleague, a friend, and he is deeply injured by the decisions not only that he had to take, but also by the assessment of the decisions that he took, that you know, it did, was it right to bomb such a place, was it right to do so. And so he lives with that, still, as he tries to grapple with life, let alone the experience of the past. In the case of when you're facing scenarios in which you either it's nearly a no when you have a responsibility to Yes, question and argue, and hold accountable those who are giving you the orders, and harass them, to the extent where they'll change their mind, which, in fact, when I was not allowed to stop the militias from rearming into training, and the UN prevented me, I didn't sort of resigned, I couldn't resign. I mean, one of the answers that we often see on Hollywood movies is I resigned in principle, well, you resigned on principle that's worth about 15 seconds on CNN, you know, you'll be replaced by somebody else who may or may not be able to do the job, God knows what if those who were with you are still loyal to you and still believe in what you're doing, then you stay and you fight and argue with the superiors until you wear them down and win, which ultimately, we were able to do, but by the time we did it, the other side that already implemented its plans. And the other thing is, is that when you do have an immoral order, from a legal authority, then it doesn't permit you to do an immoral act. When I was ordered to withdraw my myself and my my mission, and abandon Rawandans, by the Secretary General, personally, I refused it. Because although it was a legal order, it was immoral, because by then we had about 32,000 Rwandans under our protection, and one country had pulled out early, and 4000 people were killed within hours. So those 32 would have been added to the others. And so it may be legal, but it can be immoral. In such cases, you are not allowed to be immoral. However, I then became a rogue commander. And then I had an ethical problem of whether or not troops could follow my orders. And they had to volunteer to do that, in order for them to recognize that they were following a rogue commander, but in a proper ethical framework of decision making. So it has a lot of ramifications, of course. But as generals often do, they'll never be satisfied with the resources you'd give them. They'll always want more. Why? Because ultimately, they're the ones who are giving the orders of life and death, not the politician. They're the one who look people in the eye, who see and hear and smell and feel those who are suffering from it. They are the ones who are the one to must hold the moral high ground, and so much of these decisions are often politically or questionable. Yeah. I put a lot of emphasis on the generals, but I think the diplomats are in the same same similar boat to thank you.

54:36

Moderator 2: Thank you for that. Thank you for the question XXXX and also the response in general, General, I know that we're at the top of the hour, and I'm wondering if you have a few more moments that you could spend with us

RD: two more students okay.

Moderator 2: And we've you've named a lot about generations without borders and shifting into some of the things related to Setting a future context and how leaders of tomorrow can can shape things and also utilize tools at their disposal social media and other things and how to use it responsibly. So I'm mindful of those kinds of kinds of concepts. And some XXXXX, I'm wondering if you have any questions that allude to that or or in that domain?

Studen 4: Yeah, thank you so much, that, and thank you so much General delaire. For for your time today. And the additional opportunity to ask a question, I did want to ask about the generation without borders And if you've seen any specific examples, of of contexts online, where social media or other platforms have meaningfully impacted moral and ethical changes for young people, and and their their framework? And if so, if you could provide a couple of examples?

RD: I think that that is a very good question. Because the whole use of this extraordinary force multiplier, if I can use a near military term that the Social Media Communications revolution provides you is still a very immature means of making change. Immature, in the sense that it's still looking for its true focus on moving the yardsticks in subject matters in arenas that are of great essence. There have been attempts, I think that the Tunisia was a perfect example of the social media and our generation, the youth, doing any extraordinary amount of work, to try to change things. I also see scenarios where it failed. Give me Give me an example. There was a Joseph Kony was the Lord's Resistance Army in Uganda, child soldiers, massively abusing human rights of the population, and so on. And so the Lord's Resistance Army was making havoc in that whole Central African arena. And so the university students from nearly 400, universities got involved in a campaign to coalesce the youth, not only financially, but by their involvement into going after Kony. And finally getting arrested and stopping the slaughtering, and the abusing and the raping, and so on, and the use of children as weapons of war. And, and so they said, we're by this campaign, we're going to catch them, we're going to stop them, and we're going to stop this this movement. Ultimately, they were able to get something like $72 million, and they had a lot of involvement. But they failed in catching him, it was just a bridge too far for them to be able to achieve. And so what it did, however, is made Joseph Kony more known to the world, it made them more famous, and the guy gained stature in evil world in the dark side, by by this this action. So it is in my opinion, in the hands of the youth, as they maneuver with it, to try to grasp what are in fact, the scope of the of the capabilities that this instrument provides them in bringing about significant change in even revolutionary change into the societies in which we live. And I think that is growing, I think that the that the youth generation is is realizing the significant impact that it can have. And that it can coalesce and ultimately build something new. You know, we're going into an election. The population, around 25 to 28 voting population is about 2.5 million who've never voted with with this instrument of communication. You can coalesce and build political parties overnight. You can in fact, change the face of politics, because you have the balance of power. In our democracy. The old votes will go one way or another. It's the new ones that haven't been engaged and focused on that will make the change. And so I believe in things like that. Giving the right to vote at 16. We teach you what democracy is, then lets let you exercise it. And that way, you will, in fact, bring change.

1:00:15

Moderator 2: Thank you for that general and XXXXX, for your question. XXXX, you're gonna turn to you for the final question. And apologies for not getting to everyone's questions, but XXXX would you be able to offer one?

Student 13: Thank you. Yeah. Well, there's there's been so many wonderful things and interesting, profound things that my question and it does kind of pertain to some of my research interests. And it is, what would you say Is the role of spirituality in the experience of moral injury and healing from it?

RD: I'm very glad you asked that question. Because it is it is, the whole spiritual dimension of the human being is being subsumed by either religious, focus and religious I would like them to see is a constraint on the one hand, or secularism, that is essentially trying to erase the fact that you have a spiritual reference in and so a number of years ago, after Rwanda and so on, and I went to the military padres and started to discuss, you know, the impact of, of not having a reference anymore, that that our religions didn't necessarily meet some of the challenges or that there was no practicing religious dimension to our way of life anymore. And if there wasn't that, then if there's a vacuum in the spirituality, where do you turn to, you can turn to, of course, your values and your references of a society and so on. But where is the depth of your soul of your being, and where is the elements that can assist you in listening to it, and feeling it, and permitting it to sustain you. And I think that the whole dimension of spirituality is is is lost on us. My experience in Africa demonstrated to me that they the Africans gave me the sub Sahara, like everything they gave me, particularly, I sense that they are far deeper in the inner understanding of themselves. And that their, their, the depth of their, of the communications they have with their being with their past, and with the spirits there was so profound that it it does sustain them. It does sustain, where we have lost that we're visual, we're materialistic. And we're technical. And at times, trying to bring an intellectual rigor to often subjects that are fighting, that that intellectual rigor, I felt that they could handle the depth of deprivation, and adjust, ultimately, to being able to go beyond it to even bring a reconciliation, in depth that I don't think we can, I don't think, and the example I'll use here in Canada is is that in 2009, it was the 200 and 50th anniversary of the Battle of the Plains of Abraham, between the British and the French. I was trying to convince the politicals at all levels, municipal, all the way up to the federal to foreign minister, that it was important for us to commemorate the sacrifices of the past on that battlefield. And the impact it had, of course, but what they did there for what they believe that for the Aboriginal people, the First Nations people, they the French, the British soldiers, the Canadians militia, the population in Quebec and so on that suffered through that. Not one politician wanted to touch it, because they felt that they couldn't reach a simply a recognition of that event, and an acknowledgement of the sacrifices of on site. They felt it was turned into who won and who lost. To me. That was a profound sign of immaturity of our society. of not Being able to go well above that, and to show that it has depth and understanding what ultimately, people have to pay to see the future, and how we can reconcile with them, and respect them. So spirituality is an existence a lot in the African continent. And it's either subsumed by very autocratic and sometimes misogynist, very misogynist religions, or it's totally ignored. But I feel that it's a an element of the human being, that is lost in our ability to sustain the complexity and the demands of more and more of how all of humanity will be able to face the future. And that I think, is most regrettable. But hey, the fact that you're raising it is, to me a great, very positive sign.

student13: Thank you for answer.

1:06:19

Moderator 1: Yeah, thank you what we've all witnessed, and interacted in a profoundly an extraordinary third session with with great wisdom and breadth and width of topics. And the depth that is, that is extraordinary. So I think I'm speaking on behalf of all the students now that this was, this was brilliant, there was a there was highly educational and, and a learning opportunity that I hope you'll stay with, with us and with the students here for quite a while. And they will be inspired by reflecting on it or listening to it again, or another opportunity to so. So all we have a word of students, I think this is a moment for the gratitude to you again, Romeo. This was the third session. And this lines up to another credit or a webinar series that will start next week, the critical conversation series that XXXX and I building and been building over time where and this is this is really an exciting that this is prepping up towards a very exciting group conversation series that I'm looking very forward very much forward to. So without holding you up at this moment, XXXX you want to say something to then we can sort of, and I'd like the students to stay on for a little bit so that we can sort of reflect and digested a little bit further, but we want to hold you up. Yeah, go ahead.

RD: Sorry. I just tell you that I'm going to be late for the the 130 meeting. They're coming up. Okay. So do carry on, though, and I'll

Moderator 1: catch up. Yeah, yeah. Carry on.

Moderator 2: Did you have a final word before we sign off with you?

RD :Well, I hope is never going to be a final word. I hope that the debate and the discussions will continue and that you do and the generation without borders, moves, the yardsticks, moves the needle the radar, and moves us maybe generations of which I'm part of a side, because we simply can't grasp it. And that I fundamentally believe that unless we get away from this male dominated world, in all our areas, and that we become become fully male female committed, and all the gender the dimensions, that committed to the betterment of humanity, we're going to continue to fail, because so far, the men have not broken the code. And so there's got to be another solution there. So thank you very much, and I wish you well.

Moderator 1: Alright, thanks. Thanks, General. Thanks, Romeo. Okay. See you next time. Yeah. Excellent. All right. So one less that leaves us with 17. So okay, take a deep breath. It's always so exciting to see the flow of your questions and the depth that the General romeo is then responding to it. And, and, yeah, so yeah, that to me, I've seen all three of these sessions. And he really takes it to heart when you're asking and he's really sharp. And in making an effort to just give a small, small answer, but you just give given given a wide, wide answer. I don't I don't know if that's the right word to say that but maybe two sentences. So let's see, because we won't want to I don't want to hold you up way too long. Let me maybe feel 15 minutes or so, but then we can exchange some some ideas of reflections that may be helpful for all of us. Right? We sacrificed time we were with, with the, with the with, with this group together yesterday, and there may be some reflections that will help us all to get another perspective or even a more rich perspective of what we just witnessed. I'm wondering if what we can do is just ask everybody, if there's something that you took away from the conversation, one thing that you're, you're walking away with one thing that struck you, and we're going to go to those who haven't had an opportunity to ask, I'm here. So I'm going to start with them with XXXXX, XXXX, would you mind offering offering a thought from your end?

1:10:41

Student 9: Sure, um, I enjoyed all of it. I think the most profound thing that I took from it is just that it took me by surprise that his answer was love, in terms of what was the biggest part of his healing. What I can kind of see from that is that, throughout the journey of healing, whether it might take a very long time where there might be quick, the most important thing is kind of rather than thinking about problem solving, and focusing on like remediation of the problem, more emphasis on acceptance and compassion and understanding from yourself. Like the topic of self love that XXXXX mentioned, but also having a support system that also supplements that self love and that feeling of compassion and acceptance.

Moderator 2: Thank you for that XXXX. XXXXX

 Student 8: I'm gonna turn to you. Actually a little overwhelmed. I'm going to remain quiet while I digest some of this.

Moderator 2: Okay, I'll circle back. Thank you, XXXXX.

Student 6: Yeah, there was a lot, a lot of wisdom there a lot to digest. I thought it was really interesting how General delaire said a couple times about like our male dominated world, failing us. So I thought that was really interesting that he touched on that and kind of, yeah, how the current system isn't working so so well. And I thought that was encouraging coming from someone of the older generation to have that sort of view. Yeah, I don't know, I was still thinking. And I think hopefully, we'll leave all of us with a few things to continue to chew on. So thank you for that. XXXX, I want to go to you next, or to XXXXX leave us already. She laughed. And XXXX

Student 3: you know, I'm gonna have to agree with XXXX, XXXX, XXXX, there's a lot of a lot to think about. And I did like what he was saying about self love about just as open mindedness. And maybe I just want to touch on maybe one of the strengths he had as a speaker is just how engaging he was how he kind of showed how personal everything was, in terms of the causes he was talking about and championing for, I think, when he talks about the generation without borders, using social media for change, I think that's just gonna be something that's very important for us going forward is, you know, taking in a personal vested interest in what we're trying to champion for, we're trying to spread and educate others about because I mean, we can't control what others are going to think or if they're going to try and engage in these conversations. But if we don't seem like we're being genuine, or being genuine about what we're trying to champion for them, I just, you know, there's not going to be any headway being made. So I think that's something that really stuck struck me when we were talking with them. Thank you,

Moderator 2: XXXXX.

Student 11: I think I would agree with everybody saying there's still a lot to kind of take in and that I'm still thinking about it. But I agree that just this idea of having a really great support system, and self compassion and self care. And it makes me wonder what we can be doing for a lot of our individuals who are training in a post secondary training or, like I'm thinking of nurses and first responders and how do we better prepare them for some of the experiences that they're going to have going forward in their careers?

Moderator 2: Yeah, exactly. Thank you, XXXX.

Student 2: I'm also going to agree with the it's a bit much trend that some of you have talked about. To name something which hasn't been mentioned yet. I find it interesting to hear that he also talks about the possibility to go against your orders to choose what your own morality tells you. how difficult this can be, especially if you're the one who's in the field. And you have the idea that your superiors are making the right decision because they don't know what's actually going on. Of course, this is in a military setting, but I think it's something that's everybody, at some point in our life can relate to. So it gives food for thought.

Moderator 2: Yeah. Thank you, XXXX.

Student 12: Thanks. You know, as I mentioned yesterday, I work with the general extensively and still do. So I've, I've had many of these discussions, an important thing, especially when we're talking about merit moral injury, there's a couple of aspects, and it's sometimes difficult for people to understand. And he touched a little bit on this. But you know, when we use terms like moral courage, it leaves the impression that people had the courage to do the right thing. The problem with moral injury is that in a lot of cases, there is no right thing. We deal a lot with this when we're dealing with child soldiers. And now, when it comes down to the option of killing the child soldier or being killed, regardless of which way you go, it's not right. There's there's no right answer to it. And there never will be and it doesn't matter, people come and say, Well, you did the right thing. No, they killed the child. So that's, it's never going to be right. So when we counted in value, terms of right decision and wrong decision, then it's it's never going to work out well. However, it's the decision you took at that time. And your ability to refuse those orders, impacts everything. So one of the examples that we use in that, when we talk about these is in Afghanistan, there was a situation where US special forces were working with the Afghans, and there was situations where young children, young boys, specifically, were being abused by the Afghan partners that the Americans were partnered with. This was raised to higher levels. And they said, these are our partners we have to support them. So just you know, don't do anything with this. So we had one group of people who did nothing, and they went home, knowing that they allowed these children to keep being abused. Another group followed them in, went through the same thing. We're told the same thing. And then the guy said, You know what, I'm not accepting this. And he, you know, pardon expression, buddy, beat the hell out of the Afghan soldiers to protect the children was returned home, was court martialed, and dishonorably discharged, but he did the right thing to protect the children. But now he has no pension, he has that stain on his that affects his life and family for the rest. So it's great that he's saying, Hey, I did the right thing for those kids. Right. But his life has been severely impacted. He may have difficulty finding any kind of work after, right? And how does this work for his kids. So in a lot of cases, there's there's just never ever going to be a right decision. It's the decision that you make, you will always question that. And that's what leads you to the inability to forgive. And, you know, it's great to say, Oh, you should forgive yourself, you know, like the generals that he's got 800,000 voices screaming in his head all the time. So you it's very difficult to overcome. That takes a lot of time. So us, as researchers in moral injury, have to not look at it through our lens, right through our own ethical dilemmas here in the West, but to understand that the dilemmas that other people are going through, and that includes humanitarian workers who don't have enough aid to give to everybody, that includes a whole bunch of other people, that there is no right answer. So if we always counted his courage and values, we're actually creating more damage. Right, rather than trying to, you know, look at that situation. So it's a very difficult situation. And if we want to continue doing effective research in this, we really have to be willing to reach out.

 Moderator 2: Thank you for that XXXX, I noticed that you had a response. And so to XXXX, and then I want to turn to XXXXX, XXXXX, and XXXX. XXXXXX.

 Moderator 1: Now XXX, The one who didn't do anything may suffer the same moral injury by an act of omission. Right? He may have a job, but he may still suffer from the same fact that I did nothing to prevent this case. So I'm absolutely right. There's no right or wrong. But the moral issue could be related to an act of commission as well to an act of omission. Yeah, yeah, just don't want to echo that. Thank you, XXXXX.

Student 5:XXXX, I would just do your point, I would just like to say that I think it's very interesting that you would be measuring things in that way is as to how to moral injury and and moral courage could be measured. But that's a whole other discussion. So that's something to be talked about at a later time. As far as the general discussion, honestly, when he was discussing his tactile sensations of how things were impressed upon him in all of his engagements, I found that very interesting. His own personal physical responses have a huge impact on everything that he feels spiritually and emotionally and, and morally. And I found that very fascinating. So that's something I'm going to think about.

Moderator 1: XXXXX, we didn't tell the the general any direction that that the questions was going to be directed. So the fact that your question was going to was was leading him to respond to what he did was not scripted or set up. It was just the way he he thought he needed to answer you.

 Student 5: That's good. Yeah.

Moderator 2: Thank you, XXXX.

Student 7: Kind of further to the point, XXXXX, that you just made? XXXXX, I thought that question was wonderful. I thought, General delaire response was, was very profound. It gave me chills. And I think that's a good sign right there when you're getting a physical reaction to a question and a response like that. So that was very impressive.

Moderator 2: Yeah, XXXX, I want to just ask one other question of you. And that's you've, you've been to a couple of one of the other sessions and also have had a chance to, to listen to, to the other ones, you've had anything that you would say, this echoes or that he echoed, or he accentuated or anything that would be good for the group to know.

 Student 7: He was, he was a lot more direct with his responses this time. I know some of the other students had expressed, like his, his thoughts on the male dominated world. In some of his other lectures, he, he went on and on about it. And it's really wonderful to listen to him and have the opportunity to hear him talk in detail about some of these issues. And today was like a miniature version of the other ones. So he touched on on more things, just he did it in a little bit more efficient manner. So you could actually touch on all the different topics rather than just diving into one. And I feel I didn't ask question, but I mean, he touched on the stuff that I was concerned about. So yeah, It was really good.

Moderator 2: Thank you. XXXXX,

Moderator 1: XXXX you raise your hand, he wanted to say thank you, XXXX.

 Moderator 2: And then we'll go to XXXX.

Student 14: Yeah, I just wanted to add to what XXXX was saying. Because I think one of the things that I've found super interesting about what the general was saying, kind of kind of walks into this worldview and about the male dominated societies. I think what he was saying was, nations are not committing to the world or to humanity. And this rang so true to me, and he was, of course, talking about nations being conservative and in sending troops or fighting, solving things for other countries. And he talked a lot about a focus on competition, and certification and how that needs to maybe change. And this really reminded me of how we're also not really solving the problem of climate change, because we're also being so careful in implementing enough measures and focusing on which country is doing what. So I don't know. It was just a thought. It rang really true that nations are not committing to the world or to humanity. Okay,

Moderator 2: thank you.

Moderator 1: Thanks for adding that XXXX.

Moderator 2: Yeah XXXX.

Student 8: Yeah, not. Yeah. I'm trying to think of how to say this stuff diplomatically. And without bringing politics into it, by me This is apropos at least in Canada, since we're coming up on a federal election here, it occurs to me that, that we as a society need to closely examine our commitment to these efforts. So both, you know, if we're going to do something like go into Afghanistan or any other place and try to help out, then I my opinion is that we actually need to commit fully to this, partially in terms of equipping soldiers. So this is, again, this is where the politics comes in a little bit. Canada does not provide funding to the military at the same level as other developed nations, we over rely on the USA. So that's one thing to think about, at least, there's that and then I think the other piece of it is, in terms of the long term commitment. So this thing where we people about, again, I'm trying to say this in a compact way, rather than doing things that are politically expedient, and something that's, you know, in the news or whatever, and then leaving, I think we have to acknowledge the magnitude of these things. So if we're going to go in, and, and help, we may need to commit to that for generations, and acknowledge that, and I think that maybe holding politicians accountable to this, rather than saying, we're going to get in and get out, like, we need to say, either we're going to do this thing properly. Or maybe not do it, and acknowledge that as well. But make a deliberate decision one way or the other and do the thing properly.

Moderator 2: I think that there's a lot to that from, you know, we see that from a therapeutic perspective on an individual level, like, when we when we start addressing something to stay the course, in whatever way that means we see that on a national and an international and a global level. So I think, some thoughtful deliberation that is, it's important to think about terms of the consequence. Thank you. I want to turn to turn XXXX toughts that you have,

Student 9: like I stated earlier, in my experience. I'm looking at this, most of times he does witness on this mission that's been carried on around the world, he kind of think, what, what was the motive behind all these things. So let's learn from him. He had an experience with all these things, at least you get to know what goes in there, I'm so much enlightened. Because sometimes I look at all the missions that they carry out. And I cannot think of what's in front end, what's made him do these things, was it for the parroquia interest or something, because he don't kind of think you don't get any reason for them to carry out such acts, maybe trying to get 1%. And then he end up messing up the whole country, like what is happening to the Afghans now, you think the US went into to get rid of some of the Osama Bin Laden, you got a mess that they've created out of this whole country. Now the let's the Afghans, your fates, don't know what to do. Now, the same thing happens and happened in Libya as much particular about Libya, because that's a country was the prospect of Africa, the Libyans were living in good faith. And then when the real leader was killed, the whole country's name is Miss, like, if they were in hardship, those days might consider dealing to [inaudible, the whole country's messed up. So I was kind of like, what makes people or was made and what makes these higher. Authorities take these measures to deal with a certain control that I don't understand that I got a lot of matches from what he talked about. Sometimes they will be there not because of what they wish but the authorities have spoken to them to do or do other than to do certain things they need to carry out this act. But what I think the next time I get an opportunity to talk to him, maybe the the military men or the officers can also after if we try some of these things can come together and also speak to the higher authority, the UN, and then what whatever high authority that may be some of this mission, this is what they experience on the ground, because the political entities sits somewhere, give orders, they don't actually know what the things on the ground. So they should be able to speak to them after the war or after the missions that this is what begins on the ground. So that before they take up any other mission they need to go down, understand the situation before you take action, so that they don't end up creating much problem than what they anticipated.

 Moderator 2: Thank you for that. Have we missed anybody or not given anyone voice? Sorry, XXXX, go ahead.

1:30:09

Student 1: I'll try to be to be quick as we're finishing, just something to kind of build off of what general Dallaire was saying, and to sort of take a hermeneutic approach of my previous understandings and building his into that. I was thinking about Steven Hayes, therapeutic model acceptance and Commitment Therapy, and a very primary or key principle of that is psychological flexibility. And I don't think people live people have the psychological flexibility to hear difficult truths or hold distressing things, like impossible decisions ambiguity when you're forced to make these decisions. And I think that kind of results in that social betrayal in Division in these types of things. And I know a little bit what he was saying about immature, like the immaturity of our society a little bit, and then self focus sometimes. And I'll try to say this in an eloquent way, because it can be a little bit crusty, which works great when you're working with military, but I'll reference some like a thing a pastor actually said to me, he said, when we follow our own vision, and not God's vision, it causes division. And I think we can relate this to secular things as well, if we're not following the principles of human rights, of selflessness of humility, it causes division. That's where we get this, this hate and this and all these problems. So in my mind, I'm kind of not chewing on this idea of how can we grow psychological flexibility in the population and grow love, like he said, If love is a key principle and healing, again, another biblical reference, and I know XXXX left, but with spirituality, whether you're a believer or not, there's a verse in Corinthians that says, you know, if we can speak in eloquent tongues, and all these things, but we have no love, we're just clanging symbols. So if we're not doing these actions, with love, we're just clanging things, symbols, we're not actually making profound change in society, and which I think can be, you know, taken spiritually or put into secular society as well. And just basic humanitarian beliefs. So that was sort of my like, as I'm, as I'm thinking, I'm kind of thinking of psychological flexibility and love, but I really appreciate the opportunity. So that was kind of my takeaway so far, but I'm still processing like everyone else.

1:32:26

Moderator 2: Thank you for those thoughts. Thank you, everyone, for your thoughts. Um, what XXXX and I are, we're gonna close now, what will happen now is, XXXX said the has been joining us and one other one other colleague, to look at what some of the main themes were, that came up from these conversations, we may take this opportunity or this recording, if you're comfortable with that, we're going to be listening for it for some of the key themes, or if there was a quote, or something that came up from someone that might support some of the work that we do going forward with, with the with the covering of delaire series. So we may we may draw on these, if people are okay with that, we won't identify you. In anything, it will be an anonymous kind of a quote, if you're comfortable with that, we'll use it if you're not comfortable with that we're not going to use it. We also want to invite all of you to write a short reflection, just a paragraph of a reflection of what are some key takeaways that you had from today. And if you could send them to XXXX and I were putting together a book for delaire, based on all of the different covering lectures, as well as these conversations. So we wanted the the thematic analysis we're doing is actually to synthesize some of the key ideas that delaire shared with all of us. And we'll put that into an E book that will be available to all of you as well. So part of the E book is your thoughts is what comes from these conversations with him. So if you feel comfortable and want to write a reflection, you can send it into us. And we'll, we'll explore it and see if we want to include how we can include everybody's voices in this book in this compilation for something for delaire. So that he has a keepsake from this whole series going forward. And if you are not comfortable with anything that I've just said, Please let XXXXX and I know so that we can make sure that your what you've said or that anything from you is not captured in in what we're going to be doing. So just just let us know when we're by all means. We'll honor everything that you that you've said and also honor your wishes. XXXX, anything you want to close with.

Moderator 1: We've been very, very you know, you touched on everything that I felt was needed to be said. And just wanted to say the 22nd is the opening of the critical conversation series if you go to university of Leiden and then moral courage, those first words, you can you can see the whole lineup with eminent speakers from the Netherlands from Canada from the US, Australia. Rwanda, you know, nations lawyers, chaplains, indigenous people, many more. That will, we'll be engaging in a moderated discussion about a variety of topics that were embraced with what we were discussing today. It's free, invite everybody you wish to attend. It's organized when University of Alberta and Leiden University and in conjunction with the dellaire Institute, so we'll be very busy with that. Said myself, and we enjoy that it's eight hour time difference sometimes in week, late at night, when it's afternoon in Edmonton. And we do this because we think that the general has so much wisdom and experience and breath that he deserves to be heard. And all the presenters have agreed and have a personal connotation to this themselves, too. So I think it's time to end this session. And we really appreciate it just want to underscore if you want to just write it now or tonight, because then you're still in the, in the in the breast of this, just just three lines, 10 lines, whatever that that will be really appreciated. Because we can we can incorporate it into the volume that by March or so that we'll have this finished, we'll be able to share with the journal

1:36:30

Moderator 2: I put into the chat the link to the clavering and delaire lecture series, you need to be to register for each individual session so that people aren't committed to all of them. Again, it's free, we're hoping that the recordings are going to be made available on the on the Leiden website Leiden is the host University for this is the cleveringa delaire lecture series, critical conversation series. So it is driven by by their person there and so everything will be housed housed there after we finish the series. And the book will hopefully be made available. We'll know more as we go along. But we'll keep everybody informed of that as we move forward. So again, from my end, thank you, everyone, for your participating. It's just wonderful to see you all to hear your questions. I hope that it was fruitful. And we look forward to hearing your reflections and your responses, XXXX.

Moderator 1: Thank you No, much more than to add to that, than that one. So thanks for your time and your commitment. And see you next time. Okay, have a great day. Great evening, wherever you're at. Bye.

# Supplementary Figures and Tables

For more information on Supplementary Material and for details on the different file types accepted, please see [here](http://home.frontiersin.org/about/author-guidelines#SupplementaryMaterial). Figures, tables, and images will be published under a Creative Commons CC-BY licence and permission must be obtained for use of copyrighted material from other sources (including re-published/adapted/modified/partial figures and images from the internet). It is the responsibility of the authors to acquire the licenses, to follow any citation instructions requested by third-party rights holders, and cover any supplementary charges.

## Supplementary Figures

**
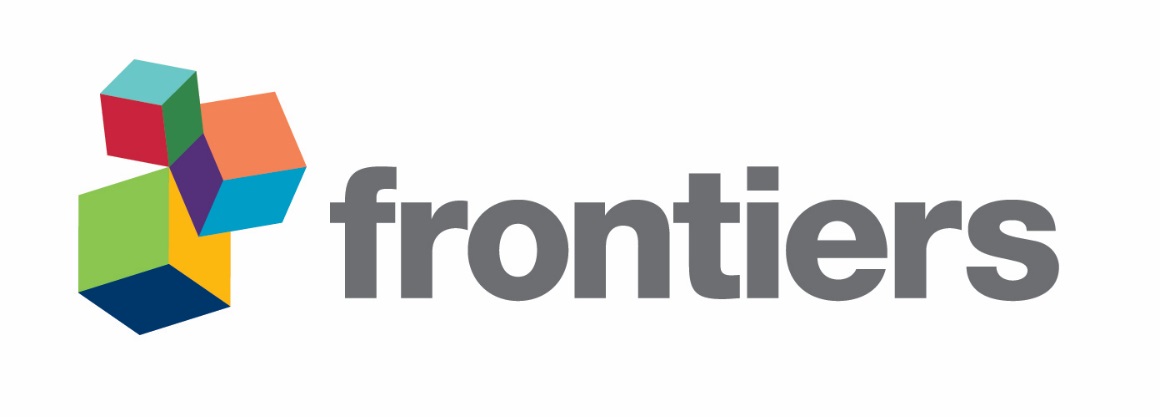
**

**Supplementary Figure 1.** The figure legends are required to have the same font as the main text, 12 point normal Times New Roman, single spaced. Please use a single paragraph for each legend and prepare the figures keeping in mind the PDF layout.
